# Caste and tobacco use: Decomposing inequalities using Global Adult Tobacco Survey, India

**DOI:** 10.1371/journal.pone.0341459

**Published:** 2026-02-11

**Authors:** Lucky Singh, Prashant Kumar Singh, Shashi Kala Saroj, Ritam Dubey, Shalini Singh

**Affiliations:** 1 Indian Council of Medical Research (ICMR), New Delhi, India; 2 Division of Preventive Oncology & Population Health, ICMR-National Institute of Cancer Prevention and Research, Noida, Uttar Pradesh, India; 3 Independent Researcher, New Delhi, India; 4 ICMR-National Institute of Cancer Prevention and Research, Noida, Uttar Pradesh, India; Indian Institute of Dalit Studies (IIDS), INDIA

## Abstract

**Background:**

India ranks second globally in tobacco consumption, with marked disparities across socially stratified caste groups. Despite tobacco control efforts, Scheduled Tribes (ST) and Scheduled Castes (SC)—historically marginalized communities—exhibit elevated tobacco use. This study examines caste-based disparities in smoked and smokeless tobacco (SLT) use and identifies factors contributing to these inequalities.

**Methods:**

We analyzed nationally representative data from the Global Adult Tobacco Survey (GATS) 2016−17, comprising 74,037 adults aged ≥15 years. Social groups were classified as ST, SC, Other Backward Classes (OBC), and General categories. We estimated prevalence rates, used multivariable logistic regression to assess associations with socio-demographic factors, and employed multivariate decomposition analysis to quantify contributors to inter-group disparities.

**Results:**

Overall tobacco use prevalence was 28.6%, with substantial variation: ST (36.0%), SC (28.9%), OBC (29.1%), and General (23.1%). SLT use was particularly high among ST (27.7%) and SC (20.7%) compared to General (16.0%). Men had 16-fold higher odds of SLT use (AOR: 16.4; 95%CI:15.2-17.7) and 23-fold higher odds of smoking [Adjusted Odds Ratio (AOR): 23.1; 95%CI:20.8-25.6] compared to women, with similar patterns within each social group. Lower education, older age, disrupted marriage, rural residence, and poor wealth status were independently associated with higher tobacco use. Decomposition analyses revealed that sex (38%), education (24%), and region (18%) explained most between-group differences, with substantial tobacco use concentrated in Eastern, Central, and Northeastern states.

**Conclusions:**

Pronounced caste-based tobacco use disparities persist in India, disproportionately affecting socioeconomically disadvantaged ST and SC populations, particularly older, less-educated in rural areas. Achieving Sustainable Development Goal 3.a requires targeted interventions addressing these structural inequalities through culturally appropriate cessation programs, enhanced health education, and region-specific tobacco control policies.

## Introduction

Tobacco consumption remains one of the leading preventable causes of morbidity and mortality worldwide, accounting for over 8 million deaths annually. Low- and middle-income countries (LMICs) bear 80% of this burden, with India alone contributing approximately 1.35 million tobacco-attributable deaths each year [1,2]. The country ranks as the world second-largest consumer and third-largest producer of tobacco products, with 28.6% (266.8 million) of adults aged ≥15 years currently using some form of tobacco [3]. Unlike many countries where cigarette smoking dominates, India tobacco landscape is characterized by diverse products: 10.7% of adults use smoked tobacco (primarily bidi—hand-rolled cigarettes made from tobacco wrapped in tendu leaves), while 21.4% consume smokeless tobacco (SLT), including *khaini*, *gutka,* betel quid with tobacco, and *paan masala* [4].

Tobacco use in India exhibits pronounced geographic and social stratification. Prevalence exceeds 40% in several northeastern states (Mizoram: 64.5%, Nagaland: 56.3%, Arunachal Pradesh: 53.2%) and tribal-majority central states (Odisha: 45.6%, Chhattisgarh: 39.1%, and Jharkhand: 38.9%), substantially surpassing the national average [3,4]. These high-prevalence regions also have large proportions of Scheduled Tribes (ST)—constitutionally recognized indigenous communities historically subjected to geographical isolation and socioeconomic marginalization. The ST population constitutes 8.6% (104 million) of India’s total population, with significant concentrations in Mizoram (94.4%), Meghalaya (86.1%), Chhattisgarh (30.6%), and Madhya Pradesh (21.1%). Similarly, Scheduled Castes (SC)—communities historically subjected to social discrimination through caste-based hierarchies—comprise 16.6% of the population and face comparable socioeconomic disadvantages [5].

While previous studies have documented overall tobacco prevalence in India and identified socioeconomic predictors of use, three critical gaps remain [6,7]. First, most research combines ST and SC populations into a single “disadvantaged” category or merges Other Backward Classes (OBC) with general populations, obscuring important heterogeneities across these constitutionally distinct groups. Second, few studies have simultaneously examined both smoked and smokeless tobacco, despite their different health consequences and distinct social patterns. Third, while descriptive differences across caste groups have been reported, the relative contribution of specific factors (demographic, socioeconomic, geographic) to these disparities remains poorly quantified. Understanding which factors most strongly drive caste-based tobacco inequalities is essential for designing effective, equity-focused interventions [8].

Addressing tobacco use disparities aligns with India’s commitments under the World Health Organization Framework Convention on Tobacco Control (WHO-FCTC) and Sustainable Development Goal (SDG) 3.a, which aims to “strengthen the implementation of the WHO-FCTC in all countries” [9]. Despite national tobacco control policies—including pictorial health warnings, advertising bans, and smoke-free place regulations—implementation remains uneven, and marginalized communities continue to experience disproportionate harm. Targeted strategies informed by empirical evidence on disparity drivers are urgently needed.

This study addresses these gaps by: (1) separately examining tobacco use patterns among ST, SC, OBC, and General caste populations using nationally representative data; (2) assessing both smoked and smokeless tobacco use; (3) identifying sociodemographic correlates within each social group; and (4) employing multivariate decomposition methods to quantify the contribution of specific factors to inter-group disparities. Our findings provide evidence to inform targeted, equity-oriented tobacco control interventions.

## Material and methods

### Data source and sampling design

This study utilized cross-sectional secondary data from the Global Adult Tobacco Survey (GATS) 2016−17 (hereafter referred to as GATS-II) for India. This survey is part of the Global Tobacco Surveillance System (GTSS), which collects multi-country data on tobacco use and associated indicators [10]. GATS-II is a nationally representative household sample survey that collected information on smoked and smokeless tobacco use. Using a multi-stage sampling design at the household level, the study collected individual-level information from 74,037 participants aged ≥15 years, including 33,772 men and 40,265 women.

Participants volunteered, and before the interview, interviewers obtained pre-informed consent from respondents, who were given the choice to withdraw or refuse to answer any question during the interview. To ensure quality, mapping, listing, and data collection work was supervised by research officers in each state/union territory. The survey was conducted by the Tata Institute of Social Sciences (TISS), Mumbai as the nodal agency, under the stewardship of the Ministry of Health and Family Welfare (MoHFW), Government of India, with collaborative funding from the World Health Organization (WHO) and the US Centers for Disease Control and Prevention (CDC) [3].

### Outcome variables

This study examined “*smoking tobacco* *use*” and “*smokeless tobacco* *(SLT) use*” as the outcome variables, representing current use by participants based on the following survey questions:

- “*Do you currently smoke tobacco? (Daily, less than daily, not at all, don’t know, refused)*”
- “*Do you currently use smokeless tobacco? (Daily, less than daily, not at all, don’t know, refused)”*

“*Daily*” and “*less-than-daily*” users were grouped as “*current tobacco users*,” while “*not at all*” and “*don’t know*” responses were grouped as “*not current tobacco users*.” No observations were found in the “*refused*” category for either form of tobacco use.

### Predictor variables

This study examined socio-economic and demographic characteristics by social categories (caste): “General,” OBC, SC, and ST. Articles 341 and 342 of the Constitution of India have notified lists of SC and ST communities, who are considered socially, educationally, and economically backward, and alienated from mainstream society due to the practice of untouchability and geographical isolation, respectively. These communities are notified under special protection to accelerate their socio-economic development [11,12].

In the GATS data, caste was self-reported in response to the question: *“Do you belong to a scheduled caste, scheduled tribe, other backward classes, or none of these groups?”* Responses of *“Don’t Know*” and “*Refused”* (n = 1,247; 1.7% of sample) were excluded from the analysis.

The socio-economic and demographic characteristics examined were: (i) age (15–18, 19–23, 24–30, 31–40, 41–50, 51–60, and over 60 years); (ii) sex (male and female); (iii) education status (no formal schooling, below primary school or primary school completed, less than secondary school completed, secondary school completed, and greater than secondary school); (iv) marital status (married, unmarried, and widowed/separated/divorced); (v) occupation (student, government employee, non-government employee, daily wage/casual laborer, self-employed, homemaker, retired/unemployed and others); (vi) religion (Hindu and Others); (vii) wealth status (poorest, poorer, middle, richer, and richest); (viii) place of residence (urban and rural); (ix) region (North, East, Central, Northeast, West, and South); (x) knowledge of adverse health effects of SLT (yes or no); (xi) knowledge of adverse health effects of smoking (yes or no). Detailed descriptions of all explanatory variables are provided in Supplementary S1 Table.

### Statistical analysis

Descriptive statistics were used to show sample characteristics by social groups (caste) in India. Bivariate analysis was used to show the prevalence of smoking, SLT use by background characteristics of the castes (Generals, OBCs, SCs, STs), and tested by the chi-square test. The multivariable logistic regression model was employed to show the adjusted association between smoked, SLT, use among the total population, General, OBC, SC, and ST social groups, separately. Logistic regression is a statistical model to assess the effect of multiple factors on a bivariate outcome, here, SLT, smoked, are two different binary outcomes, dichotomized into ‘0’ and ‘1’ for the ‘not current tobacco users’, and for the ‘current tobacco users’, respectively; and the background characteristics are explanatory variables [13].

The multivariate logit decomposition model was employed to partition the difference in the smoked and SLT use between the ST and General/ OBC social groups into component attributable to (i) group differences in predictor variables (endowments), and (ii) group differences in effects (coefficients) of these variables [14].

Mathematically, for the logistics regression model for smoked, and SLT use among the ST and General/ OBCs. The logit model for the ST and General/OBC comparison of the dependent variable in a non-linear model is a function of a linear combination of predictors and regression coefficients:


$$Y=F\left(\frac{e^{X\beta }}{1+e^{X\beta }}\right)$$

Where, *Y* denotes the *N × 1* dependent variable vector (smoked/SLT/ any tobacco), X is the *N × K* matrix of independent variables, and β is the *K × 1* vector of coefficients, and ‘F’ is the differential function mapping the logit combination of the X $\left(\frac{e^{X\beta }}{1+e^{X\beta }}\right)$ to Y. The mean difference between ‘ST and General/ OBC (Non-ST)’ social groups, labelled as ‘A’ and ‘B’, respectively, in the decomposed equation:

$\overset{―}{Y_{A}}-\overset{―}{Y_{B}}=\;\overset{―}{F\;\left(\frac{e^{X_{A}\beta_{A}}}{1+e^{X_{A}\beta_{A}}}\right)}-\overset{―}{F\left(\frac{e^{X_{B}\beta_{B}}}{1+e^{X_{B}\beta_{B}}}\right)}$


$$\underset{E}{\underbrace{\left\{\overset{―}{F\;\left(\frac{e^{X_{A}\beta_{A}}}{1+e^{X_{A}\beta_{A}}}\right)}-\overset{―}{F\left(\frac{e^{X_{B}\beta_{A}}}{1+e^{X_{B}\beta_{A}}}\right)}\right\}}}+\underset{C}{\underbrace{\left\{\overset{―}{F\;\left(\frac{e^{X_{B}\beta_{A}}}{1+e^{X_{B}\beta_{A}}}\right)}-\overset{―}{F\left(\frac{e^{X_{B}\beta_{B}}}{1+e^{X_{B}\beta_{B}}}\right)}\right\}}}$$

The first term ‘E’ denoted the part of the differential attributable to differences in endowments or characteristics, also named as ‘explained component’. Whereas, ‘C’ denoted the differential attributable to differences in coefficients or effects, named as the unexplained component or coefficient effects [14]. Here, ‘A’ is the comparison group and ‘B’ is the reference group. Estimation of the contribution to the differential would have occurred if the behavioral responses to the characteristics were fixed to the values in group A, by fixing the coefficients in the composition component to group A levels. Similarly, by fixing the B- group characteristics in the coefficient’s components, we estimated the contribution to the differential caused by the difference in effects. The decomposition for the unique contribution of each predictor in each component of the difference. For each predictor, E_*k*_ and C_*k*_, here, k is 1, 2,3 …K, that showed unique contribution of the *k*th covariate in E and C, respectively, by sequentially substituting the one group covariate with other group covariates [15].

### Sensitivity analysis

We conducted sensitivity analyses excluding the “knowledge of harmful effects” variables, as these may lie on the causal pathway from education/media exposure to tobacco use. Results are presented in Supplementary S5 Table. All analyses were conducted using Stata version 17.0 (StataCorp, College Station, TX, USA) [16]. The decomposition analyses used the *mvdcmp* command. Statistical significance was set at p < 0.05.

### Ethics statement

The study was reviewed and approved by the Institutional Ethics Committee of the ICMR–National Institute of Cancer Prevention and Research (ICMR–NICPR), Noida, India, vide letter no. NICPR/Ethics/2023 dated 9^th^ March 2023. The analysis was conducted using anonymized secondary data from the Global Adult Tobacco Survey (GATS) 2016–17, which is publicly available in the public domain. The original GATS-II obtained ethics approval from participating institutions, and informed consent was obtained from all the participants [3]. Therefore, no additional ethical approval or informed consent from participants was required.

## Results

### Sample description

This study analyzed data from 74,037 adults aged ≥15 years included in the GATS- (2016–17). The majority of respondents belonged to the OBC (45.3%), followed by General (26.2%), SC (19.1%), and ST (8.9%). Women constituted 50.5% of the ST group and approximately 48% of the other groups. Over two-fifths of participants were aged 24–40 years, with a slightly higher representation in the SC and ST categories.

Educational attainment varied considerably by social group. The proportion with no formal schooling was highest among ST (37.0%) and SC (32.8%) respondents and lowest among General (17.0%) and OBC (26.9%) respondents. Conversely, education above the secondary level was most common among the General (29.9%) and OBC (21.8%) groups. About 70% of the respondents were married, and 6–8% were with widowed, separated, or divorced status. Occupational and economic indicators reflected structural inequities. Daily wage or casual labor was predominant among ST (32.9%) and SC (30.2%) participants, whereas the General group had the lowest share (12.3%) in this category. Nearly 43% of ST, 31% of SC, 21.8% of OBC, and 13.9% of General respondents belonged to the poorest wealth quintile.

Rural residence was common across all groups (65.5%), particularly among ST (83.4%) and SC (72.9%) respondents, while urban residence was highest among the General group (45.8%). Regionally, most participants were from the Central (29.1%), Eastern (21.7%), and Southern (21.8%) regions. Although overall awareness about tobacco adverse effect was high, knowledge deficits remained pronounced among ST individuals (smoke: 49.4% and SLT: 31.2%), compared with General, OBC, and SC groups (Table 1).

**Table 1:** Descriptive statistics of the sample across different social groups, GATS 2016−17.

Background characteristics	Total		General		OBC		Scheduled Castes		Scheduled Tribes	
	n	Percent	n	percent	n	Percent	n	Percent	n	Percent
**Age (in years)**										
15-18	4641	10.47	1243	9.18	1705	10.96	856	11.01	797	10.51
19-23	7161	13.8	1887	12.31	2508	14.13	1307	14.1	1416	15.82
24-30	13867	18.18	3563	16.81	4917	17.91	2629	19.65	2641	20.13
31-40	18839	21.01	5410	21.6	6894	20.83	3380	21.04	3043	19.85
41-50	13245	15.31	3984	16.13	4987	15.13	2159	14.73	2056	15.26
51-60	8531	10.83	2626	11.56	3344	10.91	1334	10.15	1183	9.91
Over 60	7753	10.41	2569	12.41	2966	10.13	1189	9.32	992	8.52
**Sex**										
Female	40265	48.9	11854	47.55	14672	48.84	7167	49.51	6285	50.5
Male	33772	51.1	9428	52.45	12649	51.16	5687	50.49	5843	49.5
**Education**										
No formal schooling	18473	26.4	3782	17.02	7102	26.91	4216	32.76	3175	37.1
Below primary school or primary school completed	16368	20.5	3866	18.66	6034	20.29	3056	22.02	3271	23.7
Less than secondary school completed	12109	16.9	3367	17.68	4454	16.7	2069	16.77	2164	15.3
Secondary school completed	10331	14.1	3418	16.63	3884	14.27	1529	11.88	1474	10.6
Greater than secondary school	16697	22.1	6836	29.98	5834	21.79	1970	16.48	2030	13.1
**Marital status**										
Married	56984	70.1	16452	70.11	21336	70.19	9855	69	8980	71.3
Unmarried	11951	23	3370	22.94	4120	23.39	1981	22.9	2424	22
Widowed/Separated/Divorced	5102	6.9	1460	6.95	1865	6.42	1018	8.1	724	6.7
**Occupation**										
Student	6134	11.9	1859	12.32	2136	12.57	940	11.2	1180	9.3
Government employee	3355	2.7	1136	3.48	927	2.31	422	2.7	862	2.8
Non-government employee	6259	8.3	2219	10.78	2471	8.06	1018	7.5	520	4.1
Daily Wage/Casual Laborer	13749	21.2	2114	12.28	5313	20.3	3523	30.2	2693	32.9
Self-employed	13955	19.4	3971	21	5381	20.9	1732	13.4	2811	19.9
Homemaker	25833	30.1	8561	32.52	9424	29.84	4543	29	3098	25.1
Retired/Unemployed and else	4752	6.4	1422	7.62	1669	6.02	676	5.9	964	6
**Religion**										
Hindu	54015	80.34	14910	72.12	22174	79.97	11469	90.25	5172	85.84
Muslims	8785	14.22	4426	22	3946	17.68	161	0.96	143	1.43
Others	11230	5.44	1945	5.88	1200	2.35	1224	8.79	6811	12.71
Don’t know/ Not responded	7	0	1	0	1	0	–	–	2	0.01
**Wealth quintile**										
Poorest	15547	23.44	2844	13.92	5225	21.79	3239	31.07	4058	43
Poorer	18685	26.34	4201	22.31	6898	26.17	3905	31.21	3547	28.4
Middle	11278	16.79	2798	15.41	4831	18.82	2027	15.36	1579	13.9
Richer	14814	19.6	4919	24.2	5817	20.78	2473	15.35	1543	9.4
Richest	13713	13.83	6520	24.15	4550	12.45	1210	7.02	1401	5.3
**Place of residence**										
Urban	26488	34.49	9552	45.8	10462	34.5	3766	27.1	2583	16.6
Rural	47549	65.51	11730	54.2	16859	65.5	9088	72.9	9545	83.4
**Region**										
North	17128	8.7	9395	17.08	3305	3.88	4056	12.12	325	1.5
Central	11518	29.12	1850	20.43	5833	34.54	2204	29.26	1581	27.4
East	9834	21.68	2711	24.94	3573	17.4	2199	25.22	1298	26.4
North East	13574	3.71	2574	5.31	2340	1.92	1025	1.76	7450	11.4
West	7901	15.02	2818	19.31	3326	13.46	873	11.07	785	17.6
South	14082	21.76	1934	12.93	8944	28.79	2497	20.56	689	15.6
**Knowledge of adverse health effects of SLT**										
No	15510	21.89	3845	20.45	5603	20.93	2692	21.41	3173	31.2
Yes	58527	78.11	17437	79.55	21718	79.07	10162	78.59	8955	68.8
**Knowledge of adverse health effect of smoke**										
No	31243	44.07	8625	41.17	11635	44.82	5650	43.34	5037	49.4
Yes	42794	55.93	12657	58.83	15686	55.18	7204	56.66	7091	50.6
**Total**	74037	100	21282	26.23	27321	45.27	12854	19.08	12128	8.86

### Prevalence of tobacco use by social groups

Overall, 28.6% of Indian adults reported current use of any form of tobacco (smoked, smokeless, or dual), comprising 7.2% smokers, 17.9% SLT users, and 3.4% dual users. The prevalence was substantially higher among men (42.4%) than women (14.2%), with SLT being the predominant form across both sexes. Tobacco use increased progressively with age, peaking at 41.5% among those aged 51–60 years.

Marked socioeconomic and occupational gradients were evident: the prevalence exceeded 45% among daily wage/casual laborers and 43% among self-employed individuals, compared to only 4% among students. Individuals without formal education showed the highest prevalence (38.9%), followed by those with below-primary schooling (38.2%), while those educated beyond secondary level had the lowest (12.9%). Tobacco use was more prevalent among rural (32.5%) than urban residents (21.2%), and among poorest wealth quintile (41.0%) compared to the richest wealth quintile (12.4%) (Table 2).

**Table 2:** Bivariate analysis showing disparity in total (smoke/smokeless/both) tobacco use by social groups in India, 2016−17.

Background characteristics	Total			General			OBC			Scheduled Castes			Scheduled Tribes		
	Percent	95%CI		Percent	95%CI		Percent	95%CI		Percent	95%CI		Percent	95%CI	
**Age (in years)**	χ^2^p<0.001			χ^2^p<0.001			χ^2^p<0.001			χ^2^p<0.001			χ^2^p<0.001		
15-18	6.6	6.6	6.7	4.0	4.0	4.0	5.3	5.3	5.3	8.5	8.5	8.5	14.9	14.9	14.9
19-23	15.2	15.2	15.2	11.2	11.2	11.2	13.2	13.2	13.2	16.9	16.9	16.9	28.6	28.5	28.6
24-30	24.8	24.8	24.8	20.4	20.3	20.4	22.6	22.5	22.6	29.6	29.6	29.7	35.2	35.2	35.3
31-40	32.0	32.0	32.0	27.4	27.4	27.4	29.6	29.6	29.6	37.3	37.3	37.3	48.6	48.6	48.6
41-50	38.0	38.0	38.0	33.5	33.5	33.5	35.1	35.1	35.2	45.0	44.9	45.0	52.6	52.6	52.7
51-60	41.5	41.5	41.5	35.0	35.0	35.0	40.1	40.1	40.1	50.9	50.9	50.9	51.1	51.1	51.2
Over 60	41.2	41.1	41.2	34.0	34.0	34.0	40.6	40.6	40.7	50.4	50.4	50.4	52.9	52.9	52.9
**Sex**	χ^2^p<0.001			χ^2^p<0.001			χ^2^p<0.001			χ^2^p<0.001			χ^2^p<0.001		
Female	14.2	14.2	14.2	11.1	11.1	11.1	12.3	12.3	12.3	17.5	17.5	17.5	25.2	25.2	25.2
Male	42.4	42.4	42.4	37.1	37.1	37.1	40.0	40.0	40.0	49.1	49.1	49.1	56.0	56.0	56.0
**Education**	χ^2^p<0.001			χ^2^p<0.001			χ^2^p<0.001			χ^2^p<0.001			χ^2^p<0.001		
No formal schooling	38.9	38.9	38.9	36.4	36.4	36.5	35.9	35.9	35.9	43.2	43.2	43.2	45.5	45.5	45.6
Below primary school or primary school completed	38.2	38.2	38.3	34.0	34.0	34.0	36.4	36.4	36.4	41.9	41.9	41.9	49.4	49.4	49.4
Less than secondary school completed	28.7	28.7	28.7	27.1	27.1	27.1	25.1	25.1	25.1	33.5	33.4	33.5	41.6	41.6	41.7
Secondary school completed	19.9	19.9	19.9	20.6	20.5	20.6	18.5	18.5	18.5	19.6	19.6	19.7	27.2	27.2	27.3
Greater than secondary school	12.9	12.9	12.9	13.3	13.3	13.3	11.7	11.7	11.7	13.1	13.1	13.1	18.9	18.9	18.9
**Marital status**	χ^2^p<0.001			χ^2^p<0.001			χ^2^p<0.001			χ^2^p<0.001			χ^2^p<0.001		
Married	33.0	33.0	33.0	28.4	28.4	28.4	30.9	30.9	30.9	38.3	38.3	38.4	45.8	45.8	45.8
Unmarried	13.4	13.4	13.4	11.9	11.9	12.0	11.5	11.5	11.5	15.8	15.8	15.8	21.7	21.7	21.7
Widowed/Separated/Divorced	35.0	34.9	35.0	30.1	30.0	30.1	32.2	32.2	32.3	42.0	42.0	42.0	44.7	44.7	44.7
**Occupation**	χ^2^p<0.001			χ^2^p<0.001			χ^2^p<0.001			χ^2^p<0.001			χ^2^p<0.001		
Student	4.0	4.0	4.0	3.1	3.1	3.1	2.6	2.6	2.6	6.7	6.7	6.7	10.3	10.3	10.3
Government employee	23.9	23.9	23.9	23.1	23.1	23.1	22.6	22.5	22.6	21.6	21.5	21.6	37.6	37.5	37.6
Non-government employee	31.2	31.2	31.2	27.7	27.7	27.7	31.5	31.5	31.5	36.1	36.1	36.1	35.2	35.1	35.2
Daily Wage/Casual laborer	47.2	47.2	47.2	47.1	47.1	47.1	42.7	42.7	42.7	51.1	51.0	51.1	53.4	53.4	53.4
Self-employed	43.6	43.6	43.6	40.5	40.5	40.5	41.5	41.5	41.5	50.0	50.0	50.1	55.7	55.7	55.7
Homemaker	13.5	13.5	13.5	11.7	11.7	11.7	12.1	12.1	12.1	16.5	16.5	16.5	22.2	22.2	22.3
Retired/Unemployed and else	36.7	36.7	36.7	32.7	32.7	32.7	35.2	35.2	35.2	42.6	42.5	42.6	46.5	46.5	46.6
**Religion**	χ^2^p<0.001			χ^2^p<0.001			χ^2^p<0.001			χ^2^p<0.001			χ^2^p<0.001		
Hindu	28.7	28.7	28.7	23.1	23.1	23.1	26.6	26.6	26.6	34.6	34.6	34.6	40.1	40.0	40.1
Muslims	30.3	30.3	30.3	34.1	34.1	34.1	27.9	27.8	27.9	20.8	20.8	20.9	19.4	19.3	19.5
Others	22.4	22.4	22.4	10.4	10.4	10.4	12.5	12.5	12.5	23.7	23.6	23.7	45.4	45.3	45.4
**Wealth quintile**	χ^2^p<0.001			χ^2^p<0.001			χ^2^p<0.001			χ^2^p<0.001			χ^2^p<0.001		
Poorest	41.0	41.0	41.0	39.6	39.6	39.6	36.3	36.3	36.3	45.1	45.1	45.1	47.7	47.7	47.7
Poorer	33.1	33.1	33.1	31.7	31.7	31.8	30.5	30.5	30.5	36.1	36.1	36.1	41.9	41.9	41.9
Middle	27.4	27.4	27.4	26.3	26.3	26.3	27.4	27.4	27.4	27.2	27.2	27.2	31.9	31.9	31.9
Richer	20.3	20.3	20.3	20.7	20.7	20.7	19.3	19.3	19.3	20.9	20.9	20.9	25.9	25.8	25.9
Richest	12.4	12.4	12.4	12.8	12.8	12.8	11.4	11.3	11.4	11.8	11.8	11.8	21.8	21.7	21.8
**Place of residence**	χ^2^p<0.001			χ^2^p<0.001			χ^2^p<0.001			χ^2^p<0.001			χ^2^p<0.001		
Urban	21.2	21.2	21.2	19.5	19.5	19.5	19.1	19.1	19.1	28.0	27.9	28.0	32.2	32.2	32.2
Rural	32.5	32.5	32.5	29.2	29.2	29.2	30.3	30.3	30.3	35.5	35.5	35.5	42.1	42.1	42.1
**Region**	χ^2^p<0.001			χ^2^p<0.001			χ^2^p<0.001			χ^2^p<0.001			χ^2^p<0.001		
North	19.4	19.4	19.4	17.2	17.2	17.2	21.6	21.6	21.6	21.8	21.8	21.9	17.0	16.9	17.1
Central	33.5	33.5	33.5	26.3	26.3	26.3	32.0	32.0	32.0	40.5	40.4	40.5	43.0	43.0	43.0
East	33.4	33.4	33.4	29.7	29.7	29.7	28.7	28.7	28.7	39.5	39.5	39.5	46.6	46.6	46.6
North East	49.5	49.4	49.5	47.0	47.0	47.0	50.8	50.8	50.9	56.6	56.6	56.7	49.5	49.4	49.5
West	25.9	25.9	25.9	23.4	23.3	23.4	25.7	25.6	25.7	29.7	29.7	29.7	31.0	31.0	31.0
South	19.4	19.4	19.4	15.8	15.8	15.8	17.9	17.9	17.9	23.1	23.0	23.1	31.9	31.8	31.9
**Knowledge of adverse health effects of SLT**	χ^2^p<0.001			χ^2^p<0.001			χ^2^p<0.001			χ^2^p<0.001			χ^2^p<0.001		
No	35.4	35.4	35.4	29.2	29.2	29.3	33.0	33.0	33.0	39.6	39.5	39.6	49.3	49.3	49.3
Yes	26.7	26.7	26.7	23.6	23.6	23.6	24.7	24.7	24.7	31.8	31.8	31.8	36.4	36.4	36.4
**Knowledge of adverse health effects of Smoke**	χ^2^p<0.001			χ^2^p<0.001			χ^2^p<0.001			χ^2^p<0.001			χ^2^p<0.001		
No	31.7	31.7	31.7	26.3	26.3	26.3	29.4	29.4	29.4	37.4	37.4	37.4	45.4	45.4	45.4
Yes	26.2	26.2	26.2	23.7	23.7	23.7	24.1	24.1	24.1	30.5	30.4	30.5	35.6	35.6	35.6
**Total**	28.6	28.6	28.6	24.8	24.7	24.8	26.5	26.5	26.5	33.5	33.5	33.5	40.4	40.4	40.5
**N**	**74037**			**21282**			**27321**			**12854**			**12128**		

*Note*: *Note*: OBC denoted for ‘Other Backward Classes’, General denoted for ‘none of these’, and χ^2^ denotes the Chi-Square Test.

Bivariate analyses for the smoked, SLT, and both tobaccos use among social groups by their background characteristics have presented in S2 Table.

Across social groups, ST had the highest overall tobacco use (40.4%), followed by SC (33.5%), OBC (26.5%), and the General category (24.8%) individuals. SLT use mirrored this pattern—highest among ST (27.7%) and SC (20.7%)—while smoking prevalence was also greater in these groups (ST: 8.3%, SC: 8.2%) than in the General group (6.0%).

**Fig 1:**
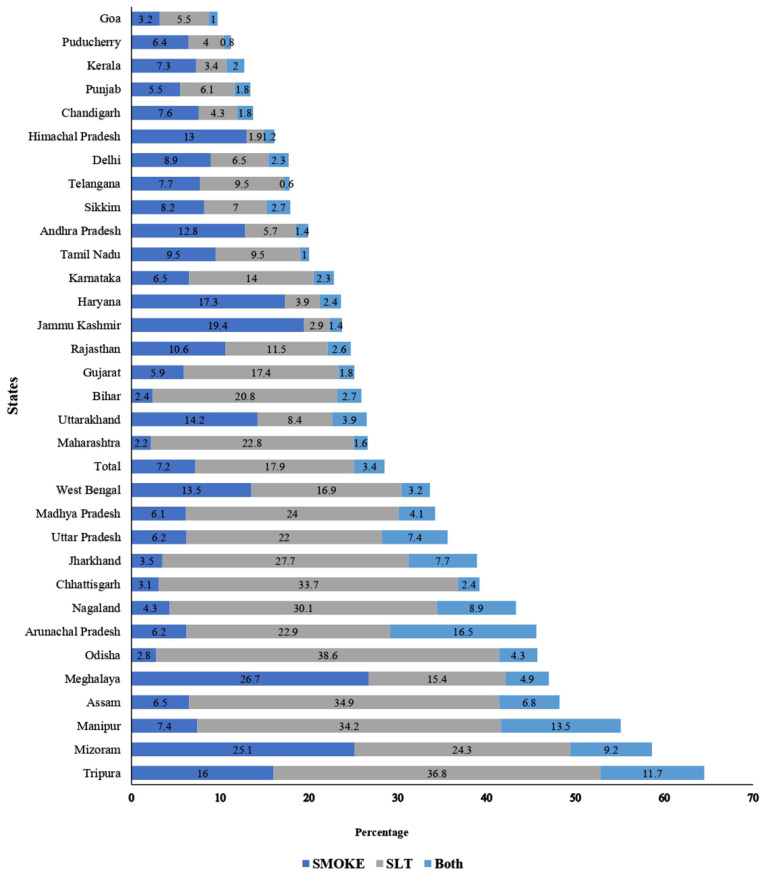
Current tobacco users (smoke, SLT, and both) by states of India, 2016−17.

**Fig 2:**
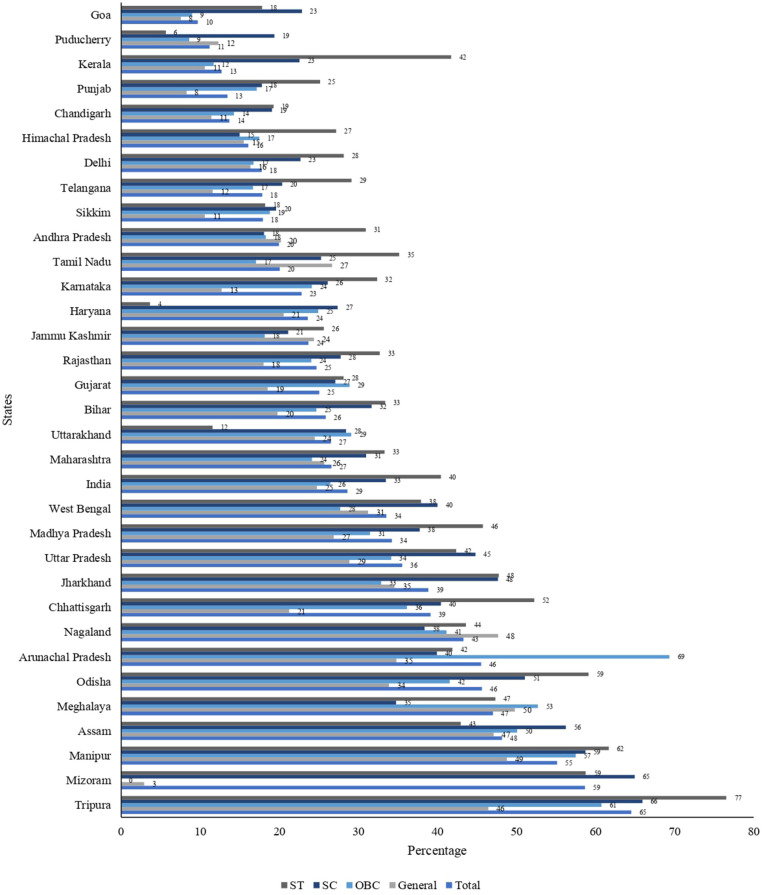
Total tobacco use among social groups in the states of India, 2016−17.

Figs 1 and 2 further reveal distinct state-level patterns, with total tobacco use exceeding 50% in the Northeastern states such as Tripura (64.5%), Mizoram (58.7%), and Manipur (55.1%), whereas Goa (9.7%) and Puducherry (11.2%) states reported the lowest levels. Fig 1 presents state-level prevalence maps for smoked tobacco, SLT, and both tobacco use. Whereas, Fig 2 shows total tobacco use prevalence by social groups inthe states of India.

More state wise information given in S3 and S4 Tables.

### Associations between socio-demographic factors and tobacco use

Multivariable analyses confirmed strong sociodemographic associations with tobacco use. Men were 5.6 times more likely to use tobacco than women [Adjusted Odds Ratio (AOR) = 5.614; 95% CI: 5.31-5.94], and odds increased consistently with age, reaching (AOR = 5.869, with 95% CI: 5.02-6.86) among those aged ≥51 years. Educational attainment showed an inverse relationship: individuals with no formal schooling were nearly three times more likely to use tobacco than those with higher education (AOR = 0.344, 95% CI: 0.320.372.84). Similar gradients were observed by wealth—poorest quintile users had 51% lesser odds than the richest (AOR: 0.494 times, 95% CI: 0.45-0.54).

Occupational status emerged as a significant determinant, with daily wage earners (AOR: 3.180, 95% C.I.: 2.77-3.65) and self-employed individuals showing markedly elevated use. Regional variation persisted even after adjustment, with residents of the Northeast (AOR = 4.71), East (AOR = 1.70), and Central (AOR = 1.70) regions having greater odds relative to the North. Awareness of tobacco’s health effects was protective, as individuals informed about the harms were 20% less likely to use tobacco (AOR = 0.80, 95% CI: 0.76–0.85).

### Group-specific associations

When stratified by caste, the ST population consistently exhibited the highest adjusted odds across all tobacco types—total tobacco use (AOR = 1.75, 95% CI: 1.63–1.88), smoked (AOR = 1.71, 95% CI: 1.49–1.96), and SLT (AOR = 1.43, 95% CI: 1.31–1.56)—relative to the General group. SC individuals also demonstrated higher odds of overall use (AOR = 1.30, 95% CI: 1.22–1.39) and SLT use (AOR = 1.33, 95% CI: 1.24–1.43), while OBCs had modestly elevated but statistically nonsignificant odds (Table 3).

**Table 3:** Adjusted multivariable binary logistic regression model for smoked, smokeless, both and overall tobacco use among the 15 and above age group, in India, 2016−17.

Background characteristics	Smoked tobacco				Smokeless tobacco				Both				Total Tobacco use			
	Odds Ratio	P-Value	95%CI		Odds Ratio	P-Value	95%CI		Odds Ratio	P-Value	95%CI		Odds Ratio	P-Value	95%CI	
**Social group (castes)**																
**General®**	1				1				1				1			
OBC	0.873***	0	0.81	0.95	1.009	0.8	0.95	1.07	1.229***	0	1.09	1.39	0.97	0.2	0.92	1.02
Scheduled Castes	1.053	0.27	0.96	1.15	1.332***	0	1.24	1.43	1.280***	0	1.11	1.48	1.304***	0	1.22	1.39
Scheduled Tribes	1.712***	0	1.55	1.9	1.435***	0	1.33	1.55	1.315***	0	1.13	1.53	1.749***	0	1.63	1.88
**Age (in years)**																
15-18^®^	1				1				1				1			
19-23	2.239***	0	1.69	2.96	1.931***	0	1.64	2.28	2.023***	0	1.44	2.85	2.375***	0	2.05	2.75
24-30	3.223***	0	2.44	4.26	2.463***	0	2.08	2.91	2.612***	0	1.86	3.68	3.387***	0	2.92	3.92
31-40	4.179***	0	3.15	5.55	3.104***	0	2.62	3.68	2.543***	0	1.8	3.6	4.473***	0	3.85	5.2
41-50	5.699***	0	4.29	7.58	3.130***	0	2.64	3.72	2.523***	0	1.78	3.59	5.236***	0	4.49	6.1
51-60	7.136***	0	5.35	9.52	3.151***	0	2.64	3.76	2.368***	0	1.65	3.39	5.869***	0	5.02	6.86
Over 60	6.393***	0	4.78	8.56	3.351***	0	2.8	4.01	1.782***	0	1.23	2.58	5.422***	0	4.62	6.36
**Sex**																
Female^®^	1				1				1				1			
Male	16.304***	0	14.43	18.42	1.398***	0	1.32	1.48	7.409***	0	6.37	8.62	5.614***	0	5.31	5.94
**Education**																
No formal schooling^®^	1				1				1				1			
Below primary school or primary school completed	0.677***	0	0.63	0.73	0.958	0.2	0.9	1.02	0.969	0.57	0.87	1.08	0.824***	0	0.78	0.87
Less than secondary school completed	0.606***	0	0.55	0.66	0.909***	0	0.85	0.97	0.780***	0	0.69	0.89	0.695***	0	0.65	0.74
Secondary school completed	0.465***	0	0.42	0.52	0.818***	0	0.75	0.89	0.547***	0	0.47	0.64	0.518***	0	0.48	0.56
Greater than secondary school	0.337***	0	0.3	0.38	0.586***	0	0.54	0.64	0.409***	0	0.34	0.49	0.344***	0	0.32	0.37
**Marital status**																
Married^®^	1				1				1							
Unmarried	0.964	0.55	0.86	1.09	0.973	0.6	0.89	1.06	0.811***	0.01	0.69	0.95	0.837***	0	0.77	0.91
Widowed/Separated/Divorced	1.223***	0	1.07	1.4	1.439***	0	1.33	1.56	1.249**	0.03	1.03	1.52	1.428***	0	1.32	1.54
**Occupation**																
Student^®^	1				1				1				1			
Government employee	1.712***	0	1.33	2.21	1.872***	0	1.55	2.27	1.757***	0	1.22	2.54	1.789***	0	1.53	2.1
Non-government employee	1.569***	0	1.23	2	2.629***	0	2.21	3.13	2.472***	0	1.76	3.47	2.389***	0	2.07	2.76
Daily Wage/Casual laborer	2.153***	0	1.71	2.72	2.672***	0	2.27	3.15	2.557***	0	1.85	3.54	3.180***	0	2.77	3.65
Self-employed	1.892***	0	1.5	2.38	2.443***	0	2.07	2.88	2.239***	0	1.62	3.1	2.559***	0	2.23	2.93
Homemaker	1.786***	0	1.38	2.32	1.451***	0	1.23	1.71	1.369*	0.09	0.95	1.97	1.581***	0	1.37	1.82
Retired/Unemployed and else	1.484***	0	1.16	1.9	1.940***	0	1.63	2.31	1.969***	0	1.4	2.78	1.821***	0	1.57	2.11
**Religion**																
Hindu^®^	1				1				1				1			
Muslims	1.284***	0	1.18	1.4	0.987	0.7	0.92	1.06	0.941	0.38	0.82	1.08	1.083***	0	1.02	1.15
Others	0.894***	0.02	0.81	0.98	0.571***	0	0.53	0.62	1.101	0.16	0.96	1.26	0.645***	0	0.6	0.69
**Wealth quintile**																
Poorest^®^	1				1				1				1			
Poorer	1.087***	0	1	1.18	0.919***	0	0.87	0.97	0.874***	0.01	0.79	0.97	0.897***	0	0.85	0.95
Middle	0.973	0.6	0.88	1.07	0.874***	0	0.81	0.94	0.772***	0	0.67	0.89	0.778***	0	0.73	0.83
Richer	0.95	0.3	0.86	1.05	0.684***	0	0.63	0.74	0.759***	0	0.65	0.88	0.661***	0	0.62	0.71
Richest	0.803***	0	0.71	0.91	0.464***	0	0.42	0.51	0.642***	0	0.53	0.78	0.494***	0	0.45	0.54
**Place of residence**																
Urban^®^	1				1				1				1			
Rural	1.136***	0	1.06	1.22	1.027	0.3	0.97	1.08	1.002	0.98	0.9	1.11	1.083***	0	1.03	1.13
**Region**																
North^®^	1				1				1				1			
Central	0.363***	0	0.33	0.4	4.823***	0	4.39	5.3	1.608***	0	1.37	1.89	1.701***	0	1.59	1.82
East	0.254***	0	0.23	0.28	5.509***	0	5.01	6.05	1.473***	0	1.25	1.74	1.702***	0	1.59	1.83
North East	0.667***	0	0.61	0.73	8.104***	0	7.37	8.91	3.659***	0	3.15	4.25	4.714***	0	4.39	5.06
West	0.225***	0	0.2	0.26	3.984***	0	3.6	4.41	0.571***	0	0.46	0.72	1.045***	0.3	0.97	1.13
South	0.537***	0	0.49	0.59	1.537***	0	1.39	1.7	0.517***	0	0.43	0.63	0.699***	0	0.65	0.75
**Knowledge of adverse health effect of smoke**																
No^®^	1				1				1				1			
Yes	1.04	0.2	0.98	1.1	_	_	_	_	0.878***	0	0.8	0.96	0.908***	0	0.87	0.95
**Knowledge of adverse health effect of SLT**																
No^®^	1				1				1				1			
Yes	_	_	_	_	0.824***	0	0.79	0.87	0.879**	0.01	0.8	0.97	0.806***	0	0.77	0.85

Note: Source: Authors’ estimation; ^®^ denotes reference category; * denotes p-values = < 0.05; ** denotes p-value = < 0.01; *** denotes p-value= < 0.001; 95% CI denotes 95% Class Interval.

Within each group, the same sociodemographic gradients persisted: older age, lower education, rural residence, and poverty were associated with higher tobacco use. Among STs, rural residency and engagement in casual or daily wage work showed the strongest associations—suggesting cumulative disadvantage. In contrast, among General and OBC groups, educational attainment and wealth gradients were more dominant, highlighting the heterogeneous nature of risk determinants across caste groups (Table 4).

**Table 4:** Adjusted multivariable binary logistic regression model of total tobacco use by social groups in India, 2016−17.

Background characteristics	General^a^				OBC^b^				Scheduled Castes^c^				Scheduled Tribes^d^			
	Odds Ratio	p-value	95%CI		Odds Ratio	p-value	95%CI		Odds Ratio	p-value	95%CI		Odds Ratio	p-value	95%CI	
**Age (in years)**																
15-18^®^	1.000				1.000				1.000				1.000			
19-23	2.864***	0.00	1.98	4.15	2.236***	0.00	1.67	2.99	2.015***	0.00	1.43	2.84	2.358***	0.00	1.86	2.99
24-30	4.873***	0.00	3.36	7.07	3.087***	0.00	2.31	4.13	2.792***	0.00	1.98	3.94	3.181***	0.00	2.49	4.06
31-40	6.354***	0.00	4.35	9.28	4.021***	0.00	3.00	5.39	3.991***	0.00	2.81	5.66	4.351***	0.00	3.38	5.60
41-50	8.526***	0.00	5.82	12.49	4.603***	0.00	3.42	6.20	5.253***	0.00	3.67	7.52	4.226***	0.00	3.25	5.49
51-60	9.029***	0.00	6.13	13.31	5.375***	0.00	3.98	7.27	6.835***	0.00	4.73	9.87	3.983***	0.00	3.03	5.24
Over 60	7.241***	0.00	4.89	10.72	5.344***	0.00	3.93	7.26	6.715***	0.00	4.60	9.80	3.401***	0.00	2.56	4.52
**Sex**																
Female^®^	1.000				1.000				1.000				1.000			
Male	7.706***	0.00	6.73	8.82	7.000***	0.00	6.32	7.75	6.800***	0.00	5.95	7.77	3.221***	0.00	2.92	3.55
**Education**																
No formal schooling^®^	1.000				1.000				1.000				1.000			
Below primary school or primary school completed	0.742***	0.00	0.65	0.84	0.748***	0.00	0.68	0.82	0.810***	0.00	0.71	0.92	1.128**	0.04	1.00	1.27
Less than secondary school completed	0.631***	0.00	0.55	0.72	0.572***	0.00	0.51	0.64	0.710***	0.00	0.61	0.83	1.026	0.72	0.89	1.18
Secondary school completed	0.474***	0.00	0.41	0.55	0.442***	0.00	0.39	0.50	0.482***	0.00	0.40	0.58	0.789***	0.00	0.67	0.93
Greater than secondary school	0.300***	0.00	0.26	0.35	0.287***	0.00	0.25	0.33	0.326***	0.00	0.27	0.40	0.555***	0.00	0.47	0.66
**Marital status**																
Married^®^	1.000				1.000				1.000				1.000			
Unmarried	0.788***	0.01	0.66	0.94	0.707***	0.00	0.61	0.82	0.818*	0.05	0.67	1.00	1.021	0.78	0.88	1.18
Widowed/Separated/Divorced	1.551***	0.00	1.32	1.82	1.592***	0.00	1.39	1.82	1.428***	0.00	1.20	1.70	1.202	0.04	1.01	1.43
**Occupation**																
Student^®^	1.000				1.000				1.000				1.000			
Government employee	2.072***	0.00	1.45	2.95	2.466***	0.00	1.74	3.49	1.667**	0.02	1.08	2.58	1.401**	0.01	1.09	1.80
Non-government employee	2.442***	0.00	1.75	3.40	3.684***	0.00	2.71	5.00	2.735***	0.00	1.87	4.01	1.659***	0.00	1.27	2.17
Daily Wage/Casual laborer	3.355***	0.00	2.41	4.67	4.290***	0.00	3.18	5.78	3.677***	0.00	2.55	5.30	3.117***	0.00	2.50	3.89
Self-employed	2.628***	0.00	1.90	3.63	3.586***	0.00	2.66	4.83	2.887***	0.00	1.99	4.19	2.287***	0.00	1.84	2.84
Homemaker	1.478***	0.03	1.05	2.08	2.418***	0.00	1.78	3.29	1.755***	0.00	1.20	2.57	1.835***	0.00	1.47	2.30
Retired/Unemployed and else	1.876***	0.00	1.33	2.65	2.674***	0.00	1.95	3.67	1.822***	0.00	1.22	2.72	1.653***	0.00	1.31	2.09
**Religion**																
Hindu^®^	1.000				1.000				1.000				1.000			
Muslim	1.104**	0.06	1.00	1.22	1.098**	0.05	1.00	1.21	0.554***	0.01	0.35	0.87	0.819	0.39	0.52	1.29
Others	0.473***	0.00	0.40	0.57	0.444***	0.00	0.36	0.55	0.643***	0.00	0.54	0.77	0.790***	0.00	0.69	0.90
**Wealth quintile**																
Poorest^®^	1.000				1.000				1.000				1.000			
Poorer	0.939	0.32	0.83	1.06	0.956	0.34	0.87	1.05	0.921***	0.19	0.82	1.04	0.748***	0.00	0.67	0.83
Middle	0.776***	0.00	0.67	0.90	0.911*	0.09	0.82	1.01	0.652***	0.00	0.56	0.76	0.686***	0.00	0.60	0.79
Richer	0.716***	0.00	0.62	0.82	0.686***	0.00	0.61	0.77	0.561***	0.00	0.47	0.66	0.690***	0.00	0.59	0.80
Richest	0.465***	0.00	0.40	0.55	0.457***	0.00	0.39	0.53	0.449***	0.00	0.35	0.57	0.730***	0.00	0.61	0.87
**Place of residence**																
Urban^®^	1.000				1.000				1.000				1.000			
Rural	1.225***	0.00	1.12	1.34	1.223***	0.00	1.13	1.32	0.983	0.77	0.88	1.10	0.846***	0.00	0.76	0.95
**Region**																
North^®^	1.000				1.000				1.000				1.000			
Central	1.534***	0.00	1.33	1.76	1.504***	0.00	1.33	1.70	1.986***	0.00	1.71	2.31	2.627***	0.00	1.84	3.75
East	1.581***	0.00	1.40	1.79	1.334***	0.00	1.16	1.53	1.975***	0.00	1.70	2.30	3.214***	0.00	2.24	4.60
North East	5.010***	0.00	4.43	5.67	3.869***	0.00	3.34	4.48	6.533***	0.00	5.44	7.85	5.378***	0.00	3.77	7.67
West	0.836***	0.01	0.73	0.95	0.986	0.84	0.86	1.14	1.524***	0.00	1.25	1.85	1.521**	0.03	1.05	2.21
South	0.619***	0.00	0.53	0.73	0.583***	0.00	0.52	0.66	0.853**	0.03	0.74	0.99	1.456*	0.05	1.00	2.13
**Knowledge of adverse health effects of smoke**																
No^®^	1.000				1.000				1.000				1.000			
Yes	0.875***	0.00	0.80	0.95	0.884***	0.00	0.82	0.95	0.834***	0.00	0.75	0.92	0.978	0.64	0.89	1.08
**Knowledge of adverse health effects of SLT**																
No^®^	1.000				1.000				1.000				1.000			
Yes	0.926	0.15	0.83	1.03	0.726***	0.00	0.67	0.79	0.813***	0.00	0.72	0.92	0.804***	0.00	0.72	0.89

Note: ^®^ denotes reference category; * denotes p-values = < 0.05; ** denotes p-value = < 0.01; *** denotes p-value= < 0.001; 95% CI denotes 95% Class Interval; a, b, c, and d denoted for separate multinomial regression analyses for each group.

More detailed association given for smoked, SLT, and both tobacco use in S6, S7 and S8 Tables, respectively.

### Decomposition of between-group disparities

The decomposition analysis quantified contributions of key determinants to the observed caste-based differences in tobacco use. The disparity in tobacco uses between STs and the General group was mainly attributable to differences in sex (2.7%), higher education (4.6%), and region of residence (18%). Wealth (6.6%) and occupational (daily wage worker/ casual labourers (6.6%) and self-employed (2.7%)) differences jointly contributed around 15%, while gaps in knowledge about health harms explained less than 5% (Table 5). Further, multivariate decomposition by smoked, SLT, and both tobacco use is given in supplementary file S9, S10, and S11 Tables, respectively.

**Table 5:** Multivariate logistic regression decomposition estimates for caste differentials in total tobacco use among ST and other social group (General and OBC) population, 2016−17.

Background characteristics	Due to Differences in Characteristics E					Due to the Difference in Coefficients C				
	Coefficient	P-value	95% CI		%	Coefficient	P-value	95% CI		%
**Age (in years)**										
15-18	1.000					1.000				
19-23	0.004	0.000	0.003	0.006	1.890	0.000	0.931	−0.003	0.003	−0.060
24-30	0.010	0.000	0.008	0.012	4.210	−0.002	0.568	−0.007	0.004	−0.740
31-40	−0.001	0.000	−0.001	0.001	−0.270	−0.001	0.812	−0.010	0.008	−0.460
41-50	−0.004	0.000	−0.005	0.003	−1.820	−0.005	0.135	−0.011	0.002	−2.130
51-60	−0.007	0.000	−0.008	0.005	−2.950	−0.005	0.020	−0.010	0.001	−2.280
Over 60	−0.008	0.000	−0.009	0.006	−3.290	−0.005	0.011	−0.010	0.001	−2.360
**Sex**										
Female	1.000					1.000				
Male	0.006	0.000	0.006	0.007	2.700	−0.037	0.000	−0.043	0.031	−16.010
**Education**										
No formal schooling	1.000					1.000				
Below primary school or primary school completed	0.002	0.046	0.000	0.003	0.660	0.009	0.000	0.006	0.012	3.800
Less than secondary school completed	0.000	0.729	0.000	0.001	0.040	0.009	0.000	0.006	0.012	3.930
Secondary school completed	0.001	0.004	0.000	0.002	0.570	0.009	0.000	0.006	0.012	3.790
Greater than secondary school	0.011	0.000	0.008	0.014	4.610	0.017	0.000	0.012	0.022	7.510
**Marital status**										
Married	1.000					1.000				
Unmarried	0.000	0.752	−0.001	0.002	0.090	0.005	0.001	0.002	0.008	2.190
Widowed/Separated/Divorced	0.000	0.035	−0.001	0.000	−0.130	−0.002	0.009	−0.003	0.000	−0.790
**Occupation**										
Student	1.000					1.000				
Government employee	0.002	0.007	0.001	0.003	0.810	−0.002	0.001	−0.004	0.001	−1.050
Non-government employee	−0.005	0.000	−0.008	0.002	−2.280	−0.006	0.000	−0.009	0.003	−2.680
Daily Wage/Casual laborer	0.015	0.000	0.012	0.018	6.590	−0.003	0.168	−0.008	0.001	−1.460
Self-employed	0.006	0.000	0.005	0.008	2.740	−0.007	0.026	−0.012	0.001	−2.890
Homemaker	−0.013	0.000	−0.018	0.008	−5.830	−0.004	0.529	−0.016	0.008	−1.640
Retired/Unemployed and else	0.002	0.000	0.001	0.002	0.670	−0.002	0.036	−0.004	0.000	−0.970
**Religion**										
Hindu	1.000					1.000				
Non-Hindu	−0.015	0.000	−0.023	0.007	−6.660	0.012	0.022	0.002	0.023	5.440
**Wealth quintile**										
Poorest	1.000					1.000				
Poorer	−0.004	0.000	−0.005	0.002	−1.550	−0.005	0.000	−0.008	0.002	−2.350
Middle	0.002	0.000	0.001	0.003	0.840	−0.003	0.011	−0.006	0.001	−1.460
Richer	0.007	0.000	0.004	0.009	2.900	0.000	0.813	−0.003	0.004	0.200
Richest	0.007	0.000	0.003	0.011	2.940	0.012	0.000	0.007	0.016	5.030
**Place of residence**										
Urban	1.000					1.000				
Rural	−0.006	0.003	−0.011	0.002	−2.790	−0.055	0.000	−0.076	0.035	−24.160
**Region**										
North	1.000					1.000				
Central	−0.005	0.000	−0.007	0.003	−2.210	0.008	0.003	0.003	0.013	3.490
East	−0.005	0.000	−0.006	0.004	−2.180	0.010	0.000	0.005	0.014	4.210
North East	0.165	0.000	0.137	0.192	71.750	0.002	0.233	−0.001	0.005	0.890
West	−0.005	0.020	−0.009	0.001	−2.100	0.006	0.008	0.002	0.011	2.670
South	−0.012	0.044	−0.023	0.000	−5.050	0.020	0.000	0.011	0.028	8.570
**Knowledge of adverse health effects of SLT**										
No	1.000					1.000				
Yes	0.003	0.000	0.002	0.004	1.260	0.001	0.872	−0.009	0.011	0.360
**Knowledge of adverse health effects of smoke**										
No	1.000					1.000				
Yes	0.000	0.673	0.000	0.000	0.000	0.006	0.078	−0.001	0.012	2.510
Overall	0.003	0.000	0.002	0.004	1.260	0.078	0.000	0.062	0.093	33.840
Constant						0.098	0.001	0.040	0.156	42.720

Notably, nearly one-third of the disparity remained unexplained, reflecting behavioral, cultural, and structural factors beyond measured variables—such as traditional practices, community norms, and access to cessation resources. A similar pattern was observed between STs and the SCs group, underscoring that structural inequity rather than individual choices predominantly drive caste-based tobacco use disparities in India (S12 Table).

## Discussion

This nationally representative study reveals profound caste-based disparities in tobacco use across India, with STs and SCs experiencing substantially higher prevalence than OBCs and General social groups . Three key patterns emerged: (1) tobacco use—particularly smokeless tobacco—is concentrated among socioeconomically disadvantaged groups, with rates exceeding 36% in ST communities compared to 23% in General populations; (2) these disparities operate through multiple mechanisms, with sex, education, and geographic region explaining approximately 80% of between-group differences; and (3) marked state-level variation suggests that cultural practices and tobacco industry targeting in high-burden regions compound structural disadvantages faced by ST and SC populations.

The elevated tobacco use among ST and SC populations reflects intersecting disadvantages operating at individual, community, and structural levels. At the individual level, these groups experience higher rates of poverty, lower educational attainment, and limited health literacy—all strong predictors of tobacco use identified in our multivariable models [17–19]. Among ST populations, 43% belonged to the poorest wealth quintile and 47% had no formal education, compared to 15% and 18%, respectively, in General populations. These disparities create conditions where tobacco—particularly inexpensive forms like bidis and smokeless products—becomes an accessible means of stress relief and social participation.

At the community level, cultural practices intersect with tobacco use in complex ways. In many tribal communities, particularly in Northeast India, offering betel quid (paan) with tobacco or serving traditional tobacco products represents hospitality and social bonding [20,21]. Women’s higher rates of SLT use in ST populations (21.3% vs. 12.3% overall) may reflect both their participation in tobacco cultivation and processing—creating routine exposure and normalization—and cultural acceptance of SLT use among tribal women, contrasting with stigma in other communities that leads to under-reporting [22,23].

At the structural level, geographic isolation of many ST communities’ limits access to health services, tobacco cessation resources, and mass media health campaigns [24–26]. Our decomposition analysis revealed that regional factors alone explained 18% of the ST-General disparity, reflecting concentrated settlement patterns in remote, underserved areas. Additionally, tobacco industry marketing strategies have historically targeted vulnerable populations, and enforcement of tobacco control regulations (display bans, age restrictions, taxation) remains weaker in remote rural areas where ST populations predominantly reside [6,27].

The pronounced male predominance in tobacco use (42.4% men vs. 14.2% women overall) deserves nuanced interpretation. While biological, social, and behavioral factors contribute to genuine sex differences, substantial evidence indicates under-reporting among women, particularly for SLT use [1,22]. Social desirability bias—where women understate socially stigmatized behaviors—is well-documented in Indian surveys. However, among ST women, where SLT use carries less stigma and has functional/occupational links, reported rates approach or exceed those of women in other groups (21.3% vs. 12.3% overall), suggesting more accurate reporting [28].

The higher male-to-female odds ratios for SLT (16.4-fold) compared to smoking (23.1-fold) may reflect both genuine differences and differential reporting bias, with SLT use more socially concealed than visible smoking [22,29]. These patterns underscore the importance of creating safe reporting environments in surveys and developing gender-sensitive tobacco control interventions.

Education emerged as the second-largest contributor (24%) to tobacco use disparities in our decomposition analysis, operating through multiple pathways. Formal education provides: (1) health literacy, enabling understanding of tobacco’s harms; (2) economic opportunities, reducing stress-driven tobacco use and enabling access to cessation resources; (3) social networks with lower tobacco normalization; and (4) exposure to health messaging through school-based programs [7,30].

The dose-response relationship we observed—with tobacco use declining progressively from no education (38% prevalence) through primary (32%), secondary (22%), to higher education (14%)—suggests that even modest educational gains could substantially reduce tobacco use. For ST communities, where 47% lack formal education, educational expansion represents a powerful long-term tobacco control strategy that simultaneously addresses broader socioeconomic disadvantages [31,32].

The concentration of high tobacco use in Eastern, Central, and Northeastern states—where ST and SC populations also cluster—reflects both compositional effects (characteristics of who lives where) and contextual effects (characteristics of places themselves) [33,34]. Historical factors include: (1) traditional cultivation of tobacco in these regions, normalizing use; (2) cultural practices incorporating tobacco products into social and ceremonial contexts; (3) lower penetration of modern tobacco control infrastructure; and (4) possible targeted marketing by tobacco companies in regions with weaker enforcement.

State-level graphs (Figs 1 and 2) reveal that even within high-burden states, tribal districts show elevated rates, suggesting community-level factors beyond state policies influence use. This geographic patterning highlights the need for region-specific interventions that account for local cultural contexts and enforcement capacities.

Our findings underscore that a “one-size-fits-all” approach to tobacco control is inadequate to address the entrenched social and regional inequities in tobacco use. While universal measures such as taxation, pictorial health warnings, and advertising bans have laid a strong policy foundation, they have not prevented widening disparities across caste and region, in India. Achieving equity in tobacco control requires context-specific, community-centered strategies integrated within broader social protection frameworks. First, culturally tailored cessation programs—implemented through trusted community health workers and tribal self-governance institutions (Gram Sabhas)—should deliver evidence-based support that acknowledges local practices and beliefs [35]. Second, targeted health education in high-burden states such as Mizoram, Nagaland, Chhattisgarh, and Odisha must use regional languages and locally accessible channels, including community health centers, agricultural extension services, and religious networks. Third, enforcement of tobacco regulations should be intensified in underserved and tribal-majority areas, with strict compliance around schools and retail points. Fourth, while taxation remains an effective population-level deterrent, equity-oriented approaches must pair it with economic assistance—such as livelihood diversification for tobacco-dependent workers and access to subsidized, non-nicotine-based cessation therapies—to minimize unintended regressive effects. Fifth, sustainable reductions in tobacco disparities demand investment in education and health literacy through strengthened school programs, particularly by reinforcing and operationalizing the TOFIE (Tobacco-Free Educational Institution) guidelines nationwide [36]. Lastly, disaggregated surveillance of tobacco use by caste and region is essential to monitor equity impacts and enable mid-course policy adjustments. Collectively, these measures can reorient national tobacco control toward inclusive, socially just, and sustainable outcomes [6,37].

## Strengths and limitations

This study’s major strengths include use of nationally representative data with robust sampling design, separate analysis of constitutionally recognized social groups often combined in previous research, examination of both smoked and smokeless tobacco, comprehensive state-level geographic analysis, and application of decomposition methods to quantify disparity contributors. The large sample size enabled subgroup analyses with adequate precision.

Several limitations warrant consideration. First, the cross-sectional design precludes causal inference; while we identify associations and quantify contributing factors, longitudinal studies are needed to establish temporal relationships. Second, self-reported tobacco use may involve under-reporting, particularly among women and in communities where use is stigmatized; biochemical validation would strengthen confidence [28]. Third, while we excluded “Don’t Know/Refused” caste responses (1.7% of sample), this may introduce selection bias if these individuals differ systematically; however, sensitivity analyses suggested minimal impact. Fourth, we could not examine specific tobacco product types within broader categories, limiting specificity of recommendations. Fifth, unmeasured confounders—including mental health status, social networks, and community tobacco norms—may partially explain observed associations.

## Conclusion

This study provides comprehensive evidence of stark caste-based tobacco use disparities in India, with STs and SCs social groups—historically marginalized and socioeconomically disadvantaged communities—experiencing substantially higher prevalence of both smoked and smokeless tobacco use compared to OBCs and General populations. These disparities concentrate among older, less-educated, poor men in rural areas, particularly in Eastern, Central, and Northeastern states, and operate through multiple intersecting mechanisms including limited education, poverty, geographic isolation, and cultural context.

Decomposition analyses revealed that sex, education, and geographic region collectively explain the majority of between-group differences, identifying clear targets for intervention. However, the persistence of substantial unexplained variation suggests that unmeasured factors—including systemic discrimination, differential tobacco industry targeting, and community-level norms—also play important roles.

Achieving Sustainable Development Goal (SDG) 3.a and WHO-FCTC implementation goals requires moving beyond population-level policies toward equity-focused strategies that address the structural determinants of tobacco use disparities. Evidence-based recommendations include: culturally tailored cessation programs delivered through trusted community channels; intensive, region-specific health education campaigns in high-burden states; strengthened regulatory enforcement in underserved areas; economic support programs for tobacco-dependent communities; and expansion of educational opportunities as a long-term disparity reduction strategy.

As India continues efforts to reduce tobacco’s devastating health toll, explicit attention to how policies affect different social groups—and intentional design of interventions to reach the most disadvantaged—will be essential to ensure that progress benefits all communities equitably. The pronounced disparities documented here demonstrate that without such focused attention, tobacco control successes may paradoxically widen health inequalities.

## Supporting information

S1 Table Detailed description of the independent variables used in the analysis of the data used in the present study. (PDF)

S2 Table Bivariate analysis showing the disparity in smoked, SLT, and both tobacco across different social groups in India, GATS 2016−17. (PDF)

S3 Table Prevalence of smoke, SLT, and both, total tobacco use across the states of India, 2016−17. (PDF)

S4 Table Prevalence of smoke, SLT, both, and total tobacco use among social groups, across the states of India, 2016−17. (PDF)

S5 Table Adjusted multivariable binary logistic regression model for smoked and SLT, both and total tobacco use among the 15 and above age group, in India, 2016−17 (Without knowledge variable). (PDF)

S6 Table Adjusted multivariable binary logistic regression model of smoked tobacco by social groups in India, 2016−17. (PDF)

S7 Table Adjusted multivariable binary logistic regression model of smokeless tobacco by social groups in India, 2016−17. (PDF)

S8 Table Adjusted multivariable binary logistic regression model of both (smoked & SLT) tobaccos use by social groups in India, 2016−17. (PDF)

S9 Table Multivariate logistic regression decomposition estimates for caste differentials in smoked tobacco use among ST and Other (General/ OBC) social groups, 2016−17. (PDF)

S10 Table Multivariate logistic regression decomposition estimates for caste differentials in smokeless tobacco use among ST and Other (General/ OBC) social groups, 2016−17. (PDF)

S11 Table Multivariate logistic regression decomposition estimates for caste differentials in both tobaccos use among ST and Other social groups, 2016−17. (PDF)

S12 Table Multivariate logistic regression decomposition estimates for caste differentials in total tobacco use among ST and SC group population, 2016−17. (PDF)

## Abbreviations

GATS: Global Adult Tobacco Survey
SLT: Smokeless Tobacco
SC: Scheduled Castes
ST: Scheduled Tribes
OBC: Other Backward Classes
WHO-FCTC: World Health Organization Framework Convention on Tobacco Control
SDG: Sustainable Development Goals
AOR: Adjusted Odds Ratio
CI: Confidence Interval
