# Supplementary material for: Caste and tobacco use: Decomposing inequalities using Global Adult Tobacco Survey, India
Source: PLoS One. 2026 Feb 11;21(2):e0341459. doi: 10.1371/journal.pone.0341459 (PMC12893575; doi:10.1371/journal.pone.0341459)
Supplement: S1 Table — (PDF) [file pone.0341459.s001.pdf]

S1 Table. Detailed description of the independent variables used in the analysis of the data used in the present study

Independent variables	Description
**Demographic Variables**	
Age	Based on individuals reporting to the question inquiring about their current age who were aged between 15 to more than 60 years were categorized into '15-18, '19-23', '24-30', '31-40', '41-50', '51-60' and 'Over 60' years.
Sex	Participant's sex was categorized into male and female.
Marital status	The marital status was categorized into three categories, namely, 'Married', 'Unmarried' and 'Widowed/Separated/Divorced'.
**Socioeconomic Variables**	
Education	Defined using the highest education level attainment, respondent's education was categorized as: no formal schooling, below primary school or primary school completed, less than secondary school completed and greater than secondary school.
Occupation	The categorization of occupation was based on the respondents answer to the question confirming the type of occupation they are involved in: Student, Government employee, non-government employee, Daily wage/Casual laborer, Self-employed, Homemaker and Retired/Unemployed and else.
Religious Groups	The religious groups were categorized under Hindu, Muslim and Others (Buddhist, Sikh, Jain, others).
Household Wealth Quintile	The detailed information obtained from the participants regarding the ownership of consumable goods and other household characteristics, [11] was used for the computation of household wealth. Correspondingly, the household wealth was categorized as: poorest, poorer, middle, richer and richest.
**Knowledge awareness variables**	
Knowledge of adverse health effects of smoking	Participant's response to the questions inquiring about their knowledge and/or self-awareness on the adverse health effects of tobacco (including chronic and non-communicable diseases), were categorized into 'No' and 'Yes'. This variable assesses the participant's current level of knowledge about the adverse health effects of tobacco (smoke and smokeless) which further provide the insights to understand the gaps in the on-going tobacco control educational health and awareness programs.
**Contextual Variable**	
Region	All the 36 States and Union Territories were included in this study, they were further divided into six geographic regions based on their geographical locations and cultural variations [12] as: North Central, East, Northeast, West and South.
Place of residence	Participant's current place of residence was categorized into urban and rural.
