# Supplementary material for: Caste and tobacco use: Decomposing inequalities using Global Adult Tobacco Survey, India
Source: PLoS One. 2026 Feb 11;21(2):e0341459. doi: 10.1371/journal.pone.0341459 (PMC12893575; doi:10.1371/journal.pone.0341459)
Supplement: S2 Table — (PDF) [file pone.0341459.s002.pdf]

S2 Table. Bivariate analysis showing the disparity in smoked, SLT, and both tobacco across different social groups in India, GATS 2016-17

Background characteristics	Total			General			OBC			Scheduled Castes			Scheduled Tribes		
	Smoked	SLT	Both	Smoked	SLT	Both	Smoked	SLT	Both	Smoked	SLT	Both	Smoked	SLT	Both
**Age (in years)**	χ^2p<0.001	χ^2p<0.001	χ^2p<0.001	χ^2p<0.001	χ^2p<0.001	χ^2p<0.001	χ^2p<0.001	χ^2p<0.001	χ^2p<0.001	χ^2 p<0.001	χ^2 p<0.001	χ^2 p<0.001	χ^2p<0.001	χ^2p<0.001	χ^2p<0.001
15-18	0.9	5	0.7	1.2	2.4	0.5	0.2	4.7	0.3	1.6	5.1	1.7	1.6	12.1	1.2
19-23	1.7	11.1	2.4	2.1	6.2	2.9	1	10.4	1.8	2.5	12.8	1.6	2.6	22.4	3.6
24-30	4.7	16.3	3.8	5.5	12.8	2.1	3.6	15.3	3.7	6	17.9	5.7	5.1	26	4.1
31-40	7.3	20.7	4.1	6.7	17.5	3.2	7.1	18.7	3.9	8.1	23.8	5.3	8.5	34.7	5.4
41-50	11.2	22.6	4.2	10.3	20	3.2	11.2	19.9	4.1	12.5	27.4	5	11.7	34.7	6.3
51-60	14.2	22.9	4.4	12.8	19.2	3	13.5	21.9	4.7	16.4	29	5.5	17.7	28.7	4.7
Over 60	12.3	25.4	3.5	11.3	21.2	1.4	11.7	25.1	3.8	13.5	30.7	6.2	17.1	31.2	4.6
**Sex**	χ^2p<0.001	χ^2p<0.001	χ^2p<0.001	χ^2p<0.001	χ^2p<0.001	χ^2p<0.001	χ^2p<0.001	χ^2p<0.001	χ^2p<0.001	χ^2 p<0.001	χ^2 p<0.001	χ^2 p<0.001	χ^2p<0.001	χ^2p<0.02	χ^2p<0.02
Female	1.5	12.3	0.5	0.85	10.1	0.2	1.4	10.3	0.5	1.9	14.9	0.7	2.3	21.9	1
Male	12.8	23.4	6.3	13.13	19.4	4.6	11.6	22.5	5.9	14.3	26.4	8.4	14.4	33.7	8
**Education**	χ^2p<0.001	χ^2p<0.001	χ^2p<0.001	χ^2p<0.001	χ^2p<0.001	χ^2p<0.001	χ^2p<0.001	χ^2p<0.001	χ^2p<0.001	χ^2 p<0.001	χ^2 p<0.001	χ^2 p<0.001	χ^2p<0.001	χ^2p<0.001	χ^2p<0.001
No formal schooling	10	24.6	4.2	10.51	23.3	2.6	9.7	21.8	4.4	10.3	27.6	5.3	10.1	31.4	4.1
Below primary school or primary school completed	9.7	23.3	5.3	9.62	21.1	3.3	9.1	21.7	5.6	11	24.5	6.4	10.3	32.7	6.4
Less than secondary school completed	6.4	18.6	3.7	7.94	15.2	4	4.8	17.1	3.2	8.3	21.5	3.6	5.6	32	4.1
Secondary school completed	5.1	12.7	2.1	5.7	12.8	2.1	4.9	12.2	1.4	4.4	11.7	3.5	5.3	17.7	4.2
Greater than secondary school	3.6	7.8	1.4	4.51	7.6	1.2	3	7.6	1.1	2.9	7.8	2.4	5.1	11.4	2.3
**Marital status**	χ^2p<0.001	χ^2p<0.001	χ^2p<0.001	χ^2p<0.001	χ^2p<0.002	χ^2p<0.003	χ^2p<0.001	χ^2p<0.002	χ^2p<0.003	χ^2 p<0.001	χ^2 p<0.001	χ^2 p<0.001	χ^2p<0.001	χ^2p<0.001	χ^2p<0.001
Married	8.8	20.2	4	8.71	16.9	2.8	8.4	18.5	4	9.6	23.5	5.2	9.8	30.9	5.1
Unmarried	2.7	8.7	2.1	3.24	6.8	1.9	1.5	8.5	1.5	4.2	8.6	3	3.6	15.6	2.5
Widowed/Separated/Divorced	6.2	26.3	2.4	6.26	22.1	1.7	5.5	24.6	2.2	7.1	31.5	3.4	7.5	33.8	3.3
**Occupation**	χ^2p<0.001	χ^2p<0.001	χ^2p<0.001	χ^2p<0.001	χ^2p<0.001	χ^2p<0.001	χ^2p<0.001	χ^2p<0.001	χ^2p<0.001	χ^2 p<0.001	χ^2 p<0.001	χ^2 p<0.001	χ^2p<0.001	χ^2p<0.001	χ^2p<0.001
Student	1	2.6	0.4	1.17	1.7	0.2	0.6	1.9	1.9	1.5	4.1	1	1.8	7.2	1.3
Government employee	8.6	13.2	2.1	10.14	11.3	1.6	7.9	12.8	3.9	7.4	12.2	1.9	8.3	23.7	5.6
Non-government employee	7.7	19.3	4.2	8.67	16.6	2.4	7.4	20.2	6.2	7.2	21.3	7.6	5.8	22.5	6.9
Daily Wage/Casual Laborer	12	28.4	6.8	15.13	24.9	7	10.8	25.8	6.5	13.1	30.5	7.5	10.2	36.6	6.6
Self-employed	12.6	24.8	6.2	13.08	22.4	5	11.5	23.5	0.4	14.9	27.5	7.6	14	36	5.7
Homemaker	1.7	11.4	0.5	1.03	10.5	0.2	1.6	10	3.1	2.3	13.3	0.9	3	18.2	1
Retired/Unemployed and else	11.6	22	3.1	12.04	18.6	2	11.2	20.9	20.9	11.2	27.1	4.3	12.9	28.6	4.9
**Religion**	χ^2 p=0.157	χ^2 p=0.503	χ^2p<0.001	χ^2p<0.001	χ^2p<0.001	χ^2p<0.001	χ^2p<0.001	χ^2p<0.001	χ^2p<0.001	χ^2 p=0.095	χ^2 p=0.028	χ^2 p=0.028	χ^2 p<0.001	χ^2 p=0.058	χ^2 p=0.058
Hindu	7.1	18.2	3.5	6.68	14.1	2.3	6.6	16.7	3.2	8.4	21.3	4.9	7.6	28.3	4.1
Muslim	8.5	18.3	3.5	10.39	20.3	3.5	7.2	17.1	3.6	7.9	10.3	2.6	5.1	13.6	0.7
Others	6	13.6	2.9	3.19	5.8	1.4	2.4	7.3	2.7	6	16.2	1.5	12.3	25.3	7
**Wealth quintile**	χ^2 p<0.001	χ^2p<0.001	χ^2p<0.001	χ^2p<0.001	χ^2p<0.001	χ^2p<0.001	χ^2p<0.001	χ^2p<0.001	χ^2p<0.001	χ^2 =p<0.005	χ^2 p<0.001	χ^2 p<0.001	χ^2 p=0.579	χ^2p<0.001	χ^2p<0.001
Poorest	8	27	6	9.95	25.7	3.9	7.2	23.4	5.7	8.3	29	7.8	8.1	34.2	5.3
Poorer	8.6	21	3.6	9.87	18.6	3.3	7.5	19.5	3.5	9.4	22.9	3.7	9.2	28.4	4.3
Middle	6.9	17.4	3.1	6.89	16.4	3	6.5	17.7	3.2	7.3	16.6	3.4	8.4	20.5	2.9
Richer	6.3	12	1.9	5.58	13.4	1.7	6.2	11.2	1.8	8.1	10.4	2.4	6.7	16.3	2.8
Richest	5.1	5.9	1.4	5.34	6.1	1.4	4.8	5.5	1.1	4.5	5.9	1.4	6.9	10.2	4.6
**Place of residence**	χ^2 p<0.001	χ^2 p<0.001	χ^2 p<0.001	χ^2p<0.001	χ^2p<0.001	χ^2p<0.001	χ^2p<0.001	χ^2p<0.001	χ^2p<0.001	χ^2 =p<0.005	χ^2 p= 0.032	χ^2 p= 0.007	χ^2 p=0.368	χ^2 p<0.005	χ^2 p<0.001
Urban	5.9	12.9	2.3	6.11	11.5	1.9	5.5	11.6	2.1	6.6	18.1	3.2	7.3	21.3	3.7
Rural	7.9	20.6	4	8.29	17.9	3	7.2	19.2	3.9	8.8	21.7	5	8.5	29	4.6
**Region**	χ^2p<0.001	χ^2p<0.001	χ^2p<0.001	χ^2p<0.001	χ^2p<0.001	χ^2p<0.001	χ^2p<0.001	χ^2p<0.001	χ^2p<0.001	χ^2 p<0.001	χ^2 p<0.001	χ^2 p<0.001	χ^2p<0.001	χ^2p<0.001	χ^2p<0.001

North	12	5.2	2.2	11.62	3.8	1.8	12.5	6	3	12.2	7.4	2.2	12.4	3.9	0.7
Central	6.8	21.3	5.5	3.9	18.8	3.6	7.4	19.2	5.4	7.9	24.7	7.9	6.8	32.6	3.7
East	6.6	23.1	3.7	9.85	17.2	2.6	3.8	21.9	3	7.7	26.8	5	4.3	35.8	6.5
North East	9	32.6	7.9	7.68	33.6	5.7	5.1	35.8	10	8.8	39.8	8	14.5	25.4	9.6
West	3.5	20.8	1.7	2.55	19.3	1.5	3.8	20	1.8	3.5	24.2	2	5.3	24.5	1.2
South	8.9	9	1.5	8.9	5.2	1.7	8		1.2	9.3	12	1.7	16.1	13.3	2.4
**Knowledge of adverse health effects of smoked tobacco**	χ^2 p=0.627		χ^2p<0.001	χ^2p=0.295		χ^2p<0.001	χ^2p=0.634		χ^2p<0.001	χ^2 p=0.848		χ^2 p<0.001	χ^2 p=0.415		χ^2 p< 0.005
No	7.1		4.1	6.58		2.7	6.8		3.4	8.1		4.8	8.6		6.8
Yes	7.3		3.3	7.79		2.4	6.5		3.2	8.3		4.5	8		3.3
**Knowledge of adverse health effects of Smoke**		χ^2p<0.001	χ^2p<0.001		χ^2p<0.001	χ^2p<0.001		χ^2p<0.001	χ^2p<0.001		χ^2 p<0.001	χ^2p<0.363		χ^2 p< 0.005	χ^2p<0.001
No		22.9	3.9		17.9	2.9		21.7	3.8		26.5	4.5		31.8	5.4
Yes		16.5	3.1		14.2	2.2		15.2	2.8		19.2	4.6		25.9	3.5
**Total**	**7.20**	**17.90**	**3.40**	**7.30**	**15.00**	**2.50**	**6.60**	**16.60**	**3.30**	**8.20**	**20.70**	**4.60**	**8.30**	**27.70**	**4.40**
**N**		**74037**			**21282**			**27321**			**12854**			**12128**	
