# Supplementary material for: Caste and tobacco use: Decomposing inequalities using Global Adult Tobacco Survey, India
Source: PLoS One. 2026 Feb 11;21(2):e0341459. doi: 10.1371/journal.pone.0341459 (PMC12893575; doi:10.1371/journal.pone.0341459)
Supplement: S3 Table — (PDF) [file pone.0341459.s003.pdf]

S3 Table. Prevalence of smoke, SLT, and both, total tobacco uses across the states of India, 2016-17

States	n	Smoke			SLT			Both			Total		
		Percent	95% C.I.		Percent	95% C.I.		Percent	95% C.I.		Percent	95% C.I.	
Jammu Kashmir	2491	19.4	19.4	19.4	2.9	2.9	2.9	2.9	1.4	1.4	23.7	23.7	23.7
Himachal Pradesh	2547	13.0	12.9	13	1.9	1.9	1.9	1.9	1.2	1.2	16.1	16.1	16.1
Punjab	2513	5.5	5.5	5.5	6.1	6.1	6.1	6.1	1.8	1.8	13.4	13.4	13.4
Chandigarh	2351	7.6	7.5	7.6	4.3	4.3	4.3	4.3	1.8	1.8	13.7	13.6	13.8
Uttarakhand	2410	14.2	14.1	14.2	8.4	8.4	8.5	8.4	3.9	3.9	26.5	26.5	26.6
Haryana	2505	17.3	17.3	17.3	3.9	3.9	3.9	3.9	2.4	2.4	23.6	23.6	23.6
Delhi	2311	8.9	8.9	9	6.5	6.5	6.5	6.5	2.3	2.3	17.8	17.8	17.8
Rajasthan	3033	10.6	10.6	10.6	11.5	11.5	11.5	11.5	2.6	2.6	24.7	24.7	24.7
Uttar Pradesh	3464	6.2	6.2	6.2	22.0	22	22	22.0	7.4	7.4	35.5	35.5	35.5
Chhattisgarh	2087	3.1	3.1	3.1	33.7	33.6	33.7	33.7	2.4	2.4	39.1	39.1	39.2
Madhya Pradesh	2934	6.1	6.1	6.1	24.0	24	24	24.0	4.1	4.1	34.2	34.2	34.2
West Bengal	2920	13.5	13.5	13.5	16.9	16.8	16.9	16.9	3.2	3.2	33.5	33.5	33.5
Jharkhand	1942	3.5	3.5	3.5	27.7	27.7	27.7	27.7	7.7	7.7	38.9	38.8	38.9
Odisha	1858	2.8	2.8	2.8	38.6	38.6	38.6	38.6	4.2	4.3	45.6	45.6	45.7
Bihar	3114	2.4	2.4	2.4	20.8	20.8	20.8	20.8	2.7	2.7	25.9	25.9	25.9
Sikkim	1416	8.2	8.1	8.3	7.0	6.9	7.1	7.0	2.7	2.8	17.9	17.8	18
Arunachal Pradesh	1373	6.2	6.2	6.2	22.9	22.8	23	22.9	16.4	16.5	45.5	45.4	45.6
Nagaland	1595	4.3	4.2	4.3	30.1	30	30.2	30.1	8.9	9	43.3	43.2	43.4
Manipur	1619	7.4	7.4	7.4	34.2	34.1	34.3	34.2	13.5	13.6	55.1	55.1	55.2
Mizoram	1566	25.1	25.1	25.2	24.3	24.2	24.4	24.3	9.2	9.3	58.7	58.6	58.8
Tripura	1559	16.0	16	16	36.8	36.8	36.9	36.8	11.7	11.7	64.5	64.5	64.6
Meghalaya	1582	26.7	26.6	26.7	15.4	15.4	15.4	15.4	4.9	5	47.0	47	47.1
Assam	2864	6.5	6.5	6.5	34.9	34.9	34.9	34.9	6.8	6.8	48.2	48.1	48.2
Gujarat	2731	5.9	5.9	5.9	17.4	17.4	17.4	17.4	1.8	1.8	25.1	25	25.1
Maharashtra	3141	2.2	2.2	2.2	22.8	22.8	22.8	22.8	1.6	1.6	26.6	26.6	26.6
Goa	2029	3.2	3.2	3.3	5.5	5.4	5.5	5.5	1	1	9.7	9.6	9.7
Andhra Pradesh	1966	12.8	12.8	12.9	5.7	5.7	5.7	5.7	1.4	1.4	20.0	19.9	20
Telangana	1827	7.7	7.7	7.7	9.5	9.5	9.5	9.5	0.6	0.6	17.8	17.8	17.9
Karnataka	2714	6.5	6.5	6.5	14.0	14	14	14.0	2.3	2.3	22.8	22.8	22.8
Kerala	2186	7.3	7.3	7.3	3.4	3.4	3.4	3.4	2	2	12.7	12.7	12.7
Tamil Nadu	2915	9.5	9.5	9.5	9.5	9.5	9.6	9.5	1	1	20.0	20	20.1
Puducherry	2474	6.4	6.4	6.5	4.0	3.9	4	4.0	0.7	0.8	11.2	11.1	11.2
Total	74037	7.2	7.2	7.2	17.9	17.9	17.9	17.9	3.4	3.4	28.6	28.6	28.6
