# Supplementary material for: Caste and tobacco use: Decomposing inequalities using Global Adult Tobacco Survey, India
Source: PLoS One. 2026 Feb 11;21(2):e0341459. doi: 10.1371/journal.pone.0341459 (PMC12893575; doi:10.1371/journal.pone.0341459)
Supplement: S4 Table — (PDF) [file pone.0341459.s004.pdf]

S4 Table. Prevalence of smoke, SLT, both, and total tobacco use among social groups, across the states of India, 2016-17

States	General					OBC					Scheduled Castes					Scheduled Tribes				
	n	Smoke	SLT	Both	Total	n	Smoke	SLT	Both	Total	n	Smoke	SLT	Both	Total	n	Smoke	SLT	Both	Total
Jammu Kashmir	1876	20.5	2.7	1.3	24.4	208	13	2.9	2.2	18.1	305	15.6	3.9	1.6	21.1	81	16.8	6.5	2.3	25.6
Himachal Pradesh	1319	12.6	1.3	1.6	15.5	463	12.9	3.5	1	17.5	669	12.4	2	0.6	15	91	24.4	2.1	0.7	27.2
Punjab	1103	3	3.9	1.4	8.3	375	7.1	6.8	3.2	17.1	1026	7.6	8.4	1.8	17.8	8	25.1	0	0	25.1
Chandigarh	1281	6.4	4.1	0.9	11.4	588	7.9	4	2.3	14.3	446	10.3	5.5	3.2	19.1	33	10.2	0	9.1	19.3
Uttarakhand	1362	13.8	7	3.7	24.5	554	14.8	10.7	3.7	29.1	464	14.8	8.8	4.9	28.4	21	4	4.2	3.3	11.5
Haryana	931	16.7	1.7	2.1	20.6	743	16.7	5	3.2	24.9	771	19.4	5.9	2.1	27.4	57	3.6	0	0	3.6
Delhi	1523	9.1	5.5	1.8	16.3	374	8.5	5.3	2.9	16.7	375	8	11.2	3.4	22.7	34	16.9	11.2	0	28.2
Rajasthan	528	6.7	9.7	1.7	18	1504	11.9	9.5	2.7	24.1	541	10.4	15.2	2.2	27.8	446	11.2	17.2	4.3	32.7
Uttar Pradesh	688	3.1	21.6	4.2	28.9	1886	7	20.4	6.8	34.2	826	7.3	26.3	11.2	44.8	55	4.2	30.3	8	42.4
Chhattisgarh	167	1.3	18.5	1.5	21.2	1021	4	29.1	3	36.1	350	3	35.8	1.6	40.4	549	1.7	48.5	2	52.2
Madhya Pradesh	467	4.4	18.6	3.8	26.9	1422	5.6	21.5	4.4	31.5	487	9.2	24.6	3.9	37.8	531	6.7	36	3.1	45.7
West Bengal	1522	14.1	14.9	2.3	31.2	364	11.2	14.3	2.2	27.7	806	15.2	20.2	4.6	40	188	6.7	24.1	7.1	37.9
Jharkhand	287	5.5	21.6	7.4	34.6	885	3	24.7	5.2	32.9	270	0.9	36.2	10.5	47.7	488	4.6	32.4	10.8	47.8
Odisha	413	3.4	25.7	4.7	33.9	584	2.7	35	3.9	41.6	424	2.3	45.4	3.4	51.1	436	2.6	51.3	5.2	59.1
Bihar	489	1.5	17.7	0.5	19.8	1740	2.7	19.7	2.4	24.7	699	1.8	24.6	5.3	31.7	186	4.1	27	2.2	33.4
Sikkim	123	6.2	4.4	0	10.6	580	8.7	6.8	3.2	18.8	68	7	7.6	4.9	19.6	641	8.3	7.4	2.5	18.2
Arunachal Pradesh	91	8.3	19.1	7.4	34.8	146	2.7	34.5	32.1	69.3	85	6	23.2	10.7	39.9	991	5.9	21.2	14.8	41.9
Nagaland	10	5.5	13.3	28.9	47.7	51	6.6	31.7	2.9	41.1	42	0.9	33.7	3.8	38.4	1490	4.4	30.2	9	43.6
Manipur	605	4.2	34.6	10	48.8	351	6.1	36.4	15	57.5	83	3.6	43	12.1	58.7	579	14.2	29.7	17.8	61.7
Mizoram	7	2.9	0	0	2.9	NA	NA	33.7	0	_	4	NA	0	65	65	1555	25.3	24.4	9	58.8
Tripura	337	13.4	29.9	3.1	46.4	347	15.7	10	11.4	60.8	403	14.1	45.5	6.3	65.9	472	19	37	20.5	76.5
Meghalaya	41	13.7	24	12	49.8	78	19.5	37.3	23.2	52.7	91	19.6	9.4	5.8	34.8	1370	28	15.7	3.7	47.3
Assam	1360	7.6	34.1	5.4	47.1	787	3.7	19.9	9.1	50.1	249	7.1	40.7	8.4	56.2	352	8.7	27	7.2	42.9
Gujarat	824	3.2	14.7	0.6	18.5	1134	6.6	20.2	2.4	28.9	354	7.3	17.9	1.9	27.1	338	9.5	16	2.6	28.1
Maharashtra	1099	2.3	21.5	1.9	25.7	1281	2.4	4.9	1.5	24.1	447	1.7	27.3	2	31	310	2.2	30.9	0.2	33.3
Goa	895	2.6	4.5	0.4	7.5	911	3.3	5.9	0.7	9	72	5.3	11.9	5.6	22.9	137	4.4	10.4	3	17.8
Andhra Pradesh	337	13.6	5.1	1.5	20.2	1085	10.9	8.2	1.4	18.3	345	11.5	5.8	0.8	18.1	199	23.1	5.9	2	30.9
Telangana	189	3.2	7.6	0.7	11.6	1118	8	14.9	0.5	16.7	358	7.2	12.6	0.5	20.4	161	13.2	14.7	1.2	29.1
Karnataka	596	5.8	6.2	0.7	12.7	1444	6.6	3.2	2.7	24.1	444	6.1	17.6	2.4	26.1	225	8.7	20.6	3.1	32.4
Kerala	551	6.1	1.6	2.9	10.6	1415	7.3	8.9	1.2	11.7	184	9.9	8.4	4.2	22.5	33	18	16.7	7	41.7
Tamil Nadu	238	16.4	7.1	3.2	26.7	2020	7.8	2.4	0.4	17	590	10.8	12.6	1.9	25.3	62	24.1	8.6	2.5	35.2
Puducherry	23	12.3	0	0	12.3	1862	5.9	2.4	0.3	8.6	576	8.2	9	2.2	19.4	9	NA	5.6	0	5.6
Total	21282	7.3	15	2.5	24.8	27321	6.6	16.6	3.3	26.5	12854	8.2	20.7	4.6	33.5	12128	8.3	27.7	4.4	40.4
