# Supplementary material for: Caste and tobacco use: Decomposing inequalities using Global Adult Tobacco Survey, India
Source: PLoS One. 2026 Feb 11;21(2):e0341459. doi: 10.1371/journal.pone.0341459 (PMC12893575; doi:10.1371/journal.pone.0341459)
Supplement: S5 Table — (PDF) [file pone.0341459.s005.pdf]

S5 Table. Adjusted multivariable binary logistic regression model for smoked and SLT, both and total tobacco use among the 15 and above age group, in India, 2016-17 (**Without knowledge variable)**

Background characteristics	Smoked				SLT				Both Tobacco use				Total Tobacco use			
	Odds Ratio	p-value	95%CI		Odds Ratio	p-value	95%CI		Odds Ratio	p-value	95%CI		Odds Ratio	p-value	95%CI	
**Social group (castes)**																
General®	1				1				1				1			
OBC	0.874***	0	0.81	0.95	1.004	0.9	0.95	1.07	1.226***	0	1.08	1.39	0.96	0.15	0.91	1.01
Scheduled Castes	1.053	0.27	0.96	1.15	1.327***	0	1.24	1.42	1.276***	0	1.11	1.47	1.30***	0.00	1.22	1.38
Scheduled Tribes	1.713***	0	1.55	1.9	1.438***	0	1.33	1.55	1.319***	0	1.13	1.54	1.75***	0.00	1.63	1.88
**Age (in years)**																
15-18®	1				1				1				1			
19-23	2.24***	0	1.69	2.96	1.909***	0	1.62	2.25	1.993***	0	1.42	2.8	2.33***	0.00	2.02	2.69
24-30	3.228***	0	2.44	4.27	2.435***	0	2.06	2.88	2.567***	0	1.82	3.61	3.32***	0.00	2.87	3.84
31-40	4.186***	0	3.15	5.56	3.064***	0	2.59	3.63	2.489***	0	1.76	3.52	4.37***	0.00	3.77	5.08
41-50	5.713***	0	4.3	7.6	3.088***	0	2.6	3.67	2.463***	0	1.73	3.5	5.11***	0.00	4.39	5.95
51-60	7.144***	0	5.36	9.53	3.114***	0	2.61	3.71	2.32***	0	1.62	3.32	5.75***	0.00	4.92	6.73
Over 60	6.4***	0	4.78	8.57	3.326***	0	2.78	3.98	1.754***	0	1.21	2.54	5.34***	0.00	4.55	6.26
**Sex**																
Female®	1				1				1				1			
Male	16.332***	0	14.45	18.46	1.403***	0	1.32	1.49	7.378***	0	6.34	8.58	5.59***	0.00	5.29	5.91
**Education**																
No formal schooling®	1				1				1				1			
Below primary school or primary school completed	0.679***	0	0.63	0.73	0.949*	0.08	0.9	1.01	0.957	0.42	0.86	1.06	0.81***	0.00	0.77	0.86
Less than secondary school completed	0.609***	0	0.56	0.67	0.89***	0	0.83	0.95	0.758***	0	0.67	0.86	0.68***	0.00	0.63	0.72
Secondary school completed	0.467***	0	0.42	0.52	0.798***	0	0.74	0.87	0.53***	0	0.45	0.62	0.50***	0.00	0.47	0.54
Greater than secondary school	0.34***	0	0.3	0.38	0.571***	0	0.52	0.62	0.394***	0	0.33	0.47	0.33***	0.00	0.31	0.36
**Marital status**																
Married®	1				1				1				1			
Unmarried	0.964	0.55	0.86	1.09	0.979	0.64	0.89	1.07	0.812**	0.01	0.69	0.95	0.84***	0.00	0.78	0.91
Widowed/Separated/Divorced	1.222***	0	1.07	1.39	1.449***	0	1.34	1.57	1.258***	0.02	1.04	1.53	1.44***	0.00	1.33	1.56
**Occupation**																
Student®	1				1				1				1			
Government employee	1.712***	0	1.33	2.21	1.873***	0	1.55	2.27	1.762***	0	1.22	2.54	1.79***	0.00	1.53	2.10
Non-government employee	1.568***	0	1.23	1.99	2.646***	0	2.23	3.15	2.497***	0	1.78	3.5	2.41***	0.00	2.09	2.78
Daily Wage/Casual laborer	2.149***	0	1.7	2.71	2.687***	0	2.28	3.17	2.582***	0	1.87	3.57	3.20***	0.00	2.79	3.67
Self-employed	1.888***	0	1.5	2.38	2.46***	0	2.09	2.9	2.266***	0	1.64	3.13	2.59***	0.00	2.26	2.96
Homemaker	1.787***	0	1.38	2.32	1.46***	0	1.24	1.73	1.376*	0.09	0.96	1.98	1.59***	0.00	1.38	1.84
Retired/Unemployed and else	1.48***	0	1.16	1.89	1.966***	0	1.65	2.34	1.997***	0	1.42	2.82	1.86***	0.00	1.60	2.15
**Religion**																
Hindu®	1				1				1				1			
Muslims	1.284***	0	1.18	1.4	0.99	0.78	0.92	1.06	0.943	0.4	0.82	1.08	1.09**	0.01	1.02	1.16
Others	0.894**	0.02	0.81	0.98	0.568***	0	0.52	0.61	1.1	0.17	0.96	1.26	0.64***	0.00	0.60	0.69
**Wealth quintile**																
Poorest®	1				1				1				1			

Poorer	1.091**	0.03	1.01	1.18	0.91***	0	0.86	0.96	0.856***	0	0.77	0.95	0.88***	0.00	0.83	0.93
Middle	0.977	0.64	0.89	1.08	0.862***	0	0.8	0.92	0.756***	0	0.66	0.87	0.76***	0.00	0.71	0.81
Richer	0.954	0.36	0.86	1.06	0.673***	0	0.62	0.73	0.74***	0	0.64	0.86	0.64***	0.00	0.60	0.69
Richest	0.807***	0	0.71	0.91	0.455***	0	0.41	0.5	0.622***	0	0.51	0.76	0.48***	0.00	0.44	0.52
**Place of residence**																
Urban®	1				1				1				1			
Rural	1.136***	0	1.06	1.22	1.031	0.25	0.98	1.09	1.007	0.89	0.91	1.12	1.09	0.00	1.04	1.14
**Region**																
North®	1				1				1				1			
Central	0.363***	0	0.33	0.4	4.825***	0	4.39	5.3	1.605***	0	1.37	1.88	1.70***	0.00	1.59	1.82
East	0.255***	0	0.23	0.29	5.578***	0	5.08	6.13	1.465***	0	1.24	1.73	1.72***	0.00	1.60	1.84
North East	0.668***	0	0.61	0.74	8.294***	0	7.54	9.12	3.681***	0	3.17	4.28	4.81***	0.00	4.48	5.16
West	0.225***	0	0.2	0.26	4.052***	0	3.67	4.48	0.577***	0	0.46	0.72	1.06	0.11	0.99	1.15
South	0.538***	0	0.49	0.59	1.586***	0	1.43	1.76	0.525***	0	0.43	0.64	0.72***	0.00	0.67	0.78

Note: Source: Authors' estimation; ® denotes reference category; * denotes p-values = <0.05; ** denotes p-value = <0.01; *** denotes p-value= <0.001; 95% CI denotes 95% Class Interval
