# Supplementary material for: Caste and tobacco use: Decomposing inequalities using Global Adult Tobacco Survey, India
Source: PLoS One. 2026 Feb 11;21(2):e0341459. doi: 10.1371/journal.pone.0341459 (PMC12893575; doi:10.1371/journal.pone.0341459)
Supplement: S6 Table — (PDF) [file pone.0341459.s006.pdf]

S6 Table: Adjusted multivariable binary logistic regression model of smoked tobacco by social groups in India, 2016-17

Background characteristics	General				OBC				Scheduled Castes				Scheduled Tribes			
	Odds Ratio	p-value	95%CI		Odds Ratio	p-value	95%CI		Odds Ratio	p-value	95%CI		Odds Ratio	p-value	95%CI	
**Age (in years)**																
15-18®	1.000				1.000				1.000				1.000			
19-23	2.234	0.01	1.18	4.23	2.747***	0.01	1.36	5.55	2.208*	0.05	1.02	4.78	1.839**	0.00	1.21	2.79
24-30	3.830***	0.00	2.04	7.18	4.805***	0.00	2.38	9.69	4.183***	0.00	1.97	8.87	1.948**	0.00	1.27	2.98
31-40	4.321***	0.00	2.28	8.18	7.903***	0.00	3.91	15.98	5.543***	0.00	2.60	11.84	2.322***	0.00	1.50	3.59
41-50	6.884***	0.00	3.63	13.06	10.213***	0.00	5.04	20.71	8.118***	0.00	3.78	17.43	2.798***	0.00	1.80	4.35
51-60	7.761***	0.00	4.07	14.80	13.469***	0.00	6.63	27.37	12.255***	0.00	5.68	26.43	2.973***	0.00	1.88	4.69
Over 60	6.469***	0.00	3.37	12.41	11.422***	0.00	5.60	23.31	10.958***	0.00	5.03	23.86	3.134***	0.00	1.97	4.99
**Sex**																
Female®	1.000				1.000				1.000				1.000			
Male	24.343***	0.00	18.18	32.60	17.942***	0.00	14.28	22.55	16.478***	0.00	12.35	21.98	12.290***	0.00	10.01	15.09
**Education**																
No formal schooling®	1.000				1.000				1.000				1.000			
Below primary school or primary school completed	0.639***	0.00	0.54	0.76	0.567***	0.00	0.50	0.65	0.818**	0.02	0.69	0.97	0.792**	0.01	0.67	0.94
Less than secondary school completed	0.599***	0.00	0.50	0.72	0.4507***	0.00	0.38	0.53	0.709***	0.00	0.58	0.87	0.738**	0.00	0.60	0.90
Secondary school completed	0.471***	0.00	0.38	0.58	0.384***	0.00	0.32	0.46	0.436***	0.00	0.34	0.57	0.588***	0.00	0.46	0.75
Greater than secondary school	0.314***	0.00	0.25	0.39	0.240309	0.00	0.20	0.30	0.382***	0.00	0.29	0.51	0.540***	0.00	0.42	0.69
**Marital status**																
Married®	1.000				1.000				1.000				1.000			
Unmarried	0.815	0.11	0.64	1.05	0.913	0.47	0.71	1.17	1.128	0.42	0.84	1.51	0.988	0.91	0.80	1.22
Widowed/Separated/Divorced	1.566***	0.00	1.21	2.03	1.099	0.45	0.86	1.40	1.203	0.22	0.89	1.62	1.191	0.22	0.90	1.58
**Occupation**																
Student®	1.000				1.000				1.000				1.000			
Government employee	2.612***	0.00	1.53	4.47	1.412	0.26	0.77	2.58	3.069*	0.01	1.32	7.12	1.256	0.25	0.85	1.85
Non-government employee	2.099***	0.01	1.25	3.52	1.409	0.23	0.81	2.46	3.050**	0.01	1.37	6.77	1.302	0.23	0.85	2.00
Daily Wage/Casual laborer	2.879***	0.00	1.72	4.82	1.489	0.16	0.86	2.59	4.618***	0.00	2.11	10.10	3.237***	0.00	2.27	4.61
Self-employed	2.583***	0.00	1.56	4.28	1.453	0.18	0.84	2.52	4.054***	0.00	1.85	8.91	2.001***	0.00	1.41	2.83
Homemaker	1.990**	0.02	1.12	3.53	1.369	0.30	0.75	2.49	3.296**	0.01	1.43	7.60	3.186***	0.00	2.14	4.75
Retired/Unemployed and else	2.057***	0.01	1.21	3.49	1.471	0.18	0.83	2.60	2.574	0.02	1.14	5.84	1.093	0.65	0.74	1.61
**Religion**																
Hindu®	1.000				1.000				1.000				1.000			
Muslim	1.506***	0.00	1.32	1.73	1.036	0.62	0.90	1.19	1.06	0.86	0.56	1.99	1.241	0.52	0.64	2.40
Others	0.349***	0.00	0.26	0.46	0.301***	0.00	0.21	0.44	0.459***	0.00	0.35	0.61	1.243**	0.03	1.03	1.51
**Wealth quintile**																
Poorest®	1.000				1.000				1.000				1.000			
Poorer	1.224**	0.02	1.03	1.46	1.097	0.22	0.95	1.27	1.195*	0.06	1.00	1.43	0.710***	0.00	0.61	0.83
Middle	0.889	0.28	0.72	1.10	1.041	0.64	0.88	1.23	1.048	0.69	0.83	1.32	0.743**	0.01	0.61	0.91
Richer	0.906	0.34	0.74	1.11	1.049	0.61	0.88	1.25	1.016	0.90	0.79	1.30	0.874	0.23	0.70	1.09
Richest	0.734***	0.01	0.58	0.92	0.759	0.02	0.60	0.96	0.971	0.87	0.69	1.37	1.031	0.82	0.80	1.33
**Place of residence**																
Urban®	1.000				1.000				1.000				1.000			
Rural	1.249***	0.00	1.10	1.42	1.279***	0.00	1.14	1.44	1.262***	0.01	1.07	1.49	0.829	0.02	0.71	0.97

**Region**																
North®	1.000				1.000				1.000				1.000			
Central	0.247***	0.00	0.19	0.31	0.409***	0.00	0.34	0.49	0.372***	0.00	0.30	0.46	0.297***	0.00	0.18	0.48
East	0.390***	0.00	0.33	0.46	0.168***	0.00	0.13	0.21	0.246***	0.00	0.19	0.31	0.154***	0.00	0.09	0.26
North East	0.331***	0.00	0.27	0.40	0.427***	0.00	0.35	0.53	0.633***	0.00	0.49	0.81	1.037	0.88	0.65	1.66
West	0.154***	0.00	0.12	0.20	0.238***	0.00	0.19	0.30	0.274***	0.00	0.19	0.39	0.359***	0.00	0.21	0.61
South	0.519***	0.00	0.42	0.63	0.503***	0.00	0.43	0.59	0.489***	0.00	0.40	0.59	0.998	0.99	0.61	1.63
**Knowledge of adverse health effects of smoke**																
No®	1.000				1.000				1.000				1.000			
Yes	1.044	0.45	0.93	1.17	0.992	0.88	0.90	1.10	0.956	0.51	0.84	1.09	0.969	0.61	0.86	1.09

Note: ® denotes reference category; * denotes p-values = <0.05; ** denotes p-value = <0.01; *** denotes p-value= <0.001; 95% CI denotes 95% Class Interval
