# Supplementary material for: Caste and tobacco use: Decomposing inequalities using Global Adult Tobacco Survey, India
Source: PLoS One. 2026 Feb 11;21(2):e0341459. doi: 10.1371/journal.pone.0341459 (PMC12893575; doi:10.1371/journal.pone.0341459)
Supplement: S7 Table — (PDF) [file pone.0341459.s007.pdf]

S7 Table. Adjusted multivariable binary logistic regression model of smokeless tobacco by social groups in India, 2016-17

Background characteristics	General				OBC				Scheduled Castes				Scheduled Tribes			
	Odds Ratio	p-value	95%CI		Odds Ratio	p-value	95%CI		Odds Ratio	p-value	95%CI		Odds Ratio	p-value	95%CI	
**Age (in years)**																
15-18®	1.00				1.00				1.00				1.00			
19-23	2.52***	0.00	1.60	3.96	1.84***	0.00	1.33	2.53	1.77**	0.00	1.21	2.60	1.88***	0.00	1.43	2.47
24-30	3.67****	0.00	2.33	5.78	2.26***	0.00	1.65	3.11	1.97***	0.00	1.35	2.89	2.44***	0.00	1.85	3.23
31-40	4.79***	0.00	3.03	7.59	2.67***	0.00	1.94	3.68	2.69***	0.00	1.83	3.96	3.26***	0.00	2.45	4.35
41-50	5.27***	0.00	3.32	8.37	2.71***	0.00	1.96	3.75	2.99***	0.00	2.01	4.43	2.83***	0.00	2.11	3.81
51-60	5.40***	0.00	3.38	8.64	2.73***	0.00	1.96	3.81	3.08***	0.00	2.05	4.60	2.61***	0.00	1.92	3.56
Over 60	5.05***	0.00	3.14	8.12	3.23***	0.00	2.31	4.52	3.08***	0.00	2.03	4.63	2.60***	0.00	1.89	3.58
**Sex**																
Female®	1.00				1.00				1.00				1.00			
Male	1.77***	0.00	1.53	2.04	2.09***	0.00	1.89	2.33	1.63***	0.00	1.42	1.86	0.76***	0.00	0.68	0.84
**Education**																
No formal schooling®	1.00				1.00				1.00				1.00			
Below primary school or primary school completed	0.92	0.21	0.80	1.05	0.92	0.11	0.84	1.02	0.86	0.03	0.76	0.98	1.21***	0.00	1.08	1.37
Less than secondary school completed	0.77***	0.00	0.65	0.89	0.83***	0.00	0.74	0.93	0.89	0.12	0.75	1.03	1.28***	0.00	1.11	1.48
Secondary school completed	0.69***	0.00	0.59	0.83	0.73***	0.00	0.64	0.84	0.77*	0.01	0.62	0.93	1.18	0.05	1.00	1.40
Greater than secondary school	0.47***	0.00	0.39	0.57	0.55***	0.00	0.48	0.64	0.52***	0.00	0.41	0.65	0.87	0.14	0.72	1.05
**Marital status**										0.64	0.07	5.08				
Married®	1.00				1.00				1.00				1.00			
Unmarried	1.02	0.85	0.83	1.25	0.84**	0.04	0.72	0.99	0.86	0.20	0.69	1.08	1.1	0.25	0.94	1.28
Widowed/Separated/Divorced	1.48***	0.00	1.24	1.76	1.77***	0.00	1.54	2.03	1.49***	0.00	1.24	1.78	1.14	0.15	0.95	1.37
**Occupation**																
Student®	1.00				1.00				1.00				1.00			
Government employee	1.96***	0.00	1.23	3.12	3.09***	0.00	2.03	4.71	1.52	0.11	0.91	2.55	1.42**	0.02	1.06	1.91
Non-government employee	2.88***	0.00	1.88	4.41	4.43***	0.00	3.06	6.41	2.69***	0.00	1.74	4.19	1.63***	0.00	1.19	2.23
Daily Wage/Casual laborer	2.42***	0.00	1.59	3.70	4.99***	0.00	3.48	7.15	2.63***	0.00	1.73	4.01	1.86***	0.00	1.44	2.40
Self-employed	2.48***	0.00	1.64	3.77	4.19***	0.00	2.93	6.00	2.24***	0.00	1.47	3.46	1.96***	0.00	1.53	2.52
Homemaker	1.47*	0.08	0.96	2.25	2.73***	0.00	1.89	3.93	1.39	0.13	0.91	2.15	1.34	0.02	1.04	1.74
Retired/Unemployed and else	1.93***	0.00	1.24	3.00	3.01***	0.00	2.06	4.40	1.96**	0.00	1.24	3.09	1.75***	0.00	1.33	2.30
**Religion**																
Hindu®	1.00				1.00				1.00				1.00			
Muslim	0.83***	0.00	0.74	0.94	1.07	0.21	0.96	1.18	0.538*	0.02	0.31	0.92	0.557*	0.06	0.30	1.02
Others	0.64***	0.00	0.51	0.80	0.53***	0.00	0.41	0.68	0.984	0.89	0.80	1.21	0.671***	0.00	0.59	0.76
**Wealth quintile**																
Poorest®	1.00				1.00				1.00				1.00			
Poorer	0.84***	0.01	0.73	0.96	0.99	1.00	0.91	1.10	0.91	0.15	0.81	1.03	0.91	0.08	0.81	1.01
Middle	0.88	0.12	0.75	1.03	1.02	0.78	0.91	1.14	0.71***	0.00	0.60	0.84	0.83	0.02	0.72	0.96
Richer	0.75***	0.00	0.64	0.88	0.69***	0.00	0.61	0.80	0.55***	0.00	0.45	0.66	0.72***	0.00	0.61	0.86
Richest	0.41***	0.00	0.33	0.50	0.42***	0.00	0.35	0.51	0.41***	0.00	0.30	0.55	0.73***	0.00	0.60	0.89
**Place of residence**																
Urban®	1.00				1.00				1.00				1.00			
Rural	1.15**	0.01	1.03	1.28	1.14***	0.00	1.04	1.25	0.89	0.05	0.78	1.00	0.89	0.05	0.78	1.00
**Region**																

North®	1.00				1.00				1.00				1.00			
Central	5.65***	0.00	4.75	6.72	3.46***	0.00	2.89	4.14	4.3***	0.00	3.61	5.21	12.4***	0.00	6.42	22.39
East	5.08***	0.00	4.33	5.97	4.43***	0.00	3.68	5.36	4.89***	0.00	4.12	5.93	15.33***	0.00	7.93	27.71
North East	12.70***	0.00	10.88	14.83	6.29***	0.00	5.19	7.63	8.44***	0.00	6.96	10.39	10.43***	0.00	5.37	18.72
West	4.27***	0.00	3.62	5.03	3.15***	0.00	2.60	3.82	4.24***	0.00	3.36	5.20	6.71***	0.00	3.43	12.26
South	1.28**	0.05	1.00	1.62	1.12	0.23	0.93	1.35	1.78***	0.00	1.47	2.15	3.62***	0.00	1.82	6.72
**Knowledge of adverse health effects of SLT**																
No®	1.00				1.00				1.00				1.00			
Yes	0.94	0.34	0.85	1.06	0.69***	0.00	0.63	0.75	0.77***	0.00	0.69	0.86	0.96	0.39	0.87	1.06

Note: ® denotes reference category; * denotes p-values = <0.05; ** denotes p-value = <0.01; *** denotes p-value= <0.001; 95% CI denotes 95% Class Interval, Other caste includes:
