# Supplementary material for: Caste and tobacco use: Decomposing inequalities using Global Adult Tobacco Survey, India
Source: PLoS One. 2026 Feb 11;21(2):e0341459. doi: 10.1371/journal.pone.0341459 (PMC12893575; doi:10.1371/journal.pone.0341459)
Supplement: S8 Table — (PDF) [file pone.0341459.s008.pdf]

S8 Table. Adjusted multivariable binary logistic regression model of both (smoked & SLT) tobaccos use by social groups in India, 2016-17

Background characteristics	General				OBC				Scheduled Castes				Scheduled Tribes			
	Odds Ratio	p-value	95%CI		Odds Ratio	p-value	95%CI		Odds Ratio	p-value	95%CI		Odds Ratio	p-value	95%CI	
**Age (in years)**																
15-18®	1.00				1.00				1.00				1.00			
19-23	1.47	0.42	0.58	3.69	1.74	0.13	0.85	3.57	1.45	0.36	0.66	3.23	2.44***	0.00	1.43	4.17
24-30	1.87	0.18	0.76	4.6	2.49***	0.01	1.24	5.01	2.14*	0.06	0.98	4.68	2.79***	0.00	1.62	4.82
31-40	2.02	0.13	0.81	5.03	2.30**	0.02	1.14	4.66	2.04*	0.08	0.92	4.53	2.78***	0.00	1.60	4.85
41-50	1.93	0.16	0.77	4.85	2.40**	0.02	1.18	4.89	1.98*	0.10	0.88	4.46	2.73***	0.00	1.55	4.80
51-60	1.68	0.28	0.66	4.29	2.37**	0.02	1.15	4.87	1.76	0.18	0.77	4.04	2.66***	0.00	1.48	4.76
Over 60	1.16	0.77	0.44	3.02	1.68	0.17	0.80	3.52	2.23**	0.06	0.97	5.14	1.56	0.16	0.84	2.87
**Sex**																
Female®	1.00				1.00				1.00				1.00			
Male	21.77***	0.00	12.48	37.99	9.67***	0.00	7.05	13.26	18.31***	0.00	11.22	29.89	3.82***	0.00	3.13	4.67
**Education**																
No formal schooling®	1.00				1.00				1.00				1.00			
Below primary school or primary school completed	0.8	0.13	0.61	1.06	0.97	0.73	0.81	1.16	0.87	0.28	0.69	1.11	1.16	0.14	0.95	1.42
Less than secondary school completed	0.81	0.18	0.6	1.1	0.73***	0.01	0.59	0.92	0.67***	0.01	0.50	0.91	0.84	0.17	0.66	1.07
Secondary school completed	0.49***	0.00	0.34	0.71	0.47***	0.00	0.35	0.62	0.57***	0.01	0.38	0.85	0.68***	0.01	0.51	0.91
Greater than secondary school	0.43***	0.00	0.29	0.62	0.34***	0.00	0.25	0.47	0.39***	0.00	0.24	0.61	0.51***	0.00	0.37	0.71
**Marital status**																
Married®	1.00				1.00				1.00				1.00			
Unmarried	0.70*	0.08	0.47	1.05	0.71**	0.03	0.51	0.96	0.86	0.48	0.58	1.30	0.86	0.23	0.66	1.10
Widowed/Separated/Divorced	1.89***	0.01	1.19	3.02	1.19	0.36	0.82	1.72	1.46*	0.09	0.94	2.26	1.16	0.37	0.84	1.62
**Occupation**																
Student®	1.00				1.00				1.00				1.00			
Government employee	3.13***	0.04	1.06	9.28	2.29	0.12	0.81	6.50	1.67	0.35	0.57	4.86	1.44	0.14	0.89	2.32
Non-government employee	4.11***	0.01	1.47	11.49	5.53***	0.00	2.23	13.67	2.45*	0.06	0.98	6.14	1.74**	0.03	1.04	2.91
Daily Wage/Casual laborer	6.66***	0.00	2.4	18.45	5.18***	0.00	2.12	12.69	2.68**	0.03	1.10	6.52	1.63**	0.03	1.05	2.54
Self-employed	4.29***	0.01	1.56	11.85	5.13***	0.00	2.10	12.54	2.44*	0.05	1.00	5.97	1.42	0.11	0.92	2.20
Homemaker	2.59	0.11	0.8	8.36	2.24	0.10	0.85	5.92	2.44*	0.09	0.88	6.76	1.31	0.27	0.81	2.11
Retired/Unemployed and else	3.00**	0.04	1.03	8.73	3.98***	0.00	1.57	10.08	1.59	0.34	0.61	4.20	1.72**	0.02	1.09	2.71
**Religion**																
Hindu®	1.00				1.00				1.00				1.00			
Muslim	0.69***	0.00	0.55	0.89	1.25**	0.02	1.03	1.50	0.5	0.26	0.16	1.64	0.82	0.72	0.28	2.41
Others	1.01	0.94	0.69	1.5	1.37*	0.08	0.96	1.95	0.53**	0.02	0.31	0.91	0.96	0.67	0.77	1.18
**Wealth quintile**																
Poorest®	1.00				1.00				1.00				1.00			
Poorer	1.09	0.50	0.84	1.42	0.82**	0.04	0.69	0.99	0.79*	0.06	0.63	1.01	0.87	0.13	0.73	1.04
Middle	0.85	0.38	0.61	1.21	0.77**	0.02	0.61	0.96	0.60***	0.01	0.42	0.86	0.79*	0.07	0.62	1.02
Richer	0.89	0.53	0.64	1.26	0.63***	0.00	0.48	0.81	0.63**	0.02	0.43	0.93	0.94	0.66	0.72	1.23
Richest	0.64**	0.03	0.42	0.96	0.58***	0.00	0.40	0.84	0.47**	0.02	0.25	0.89	0.84	0.28	0.61	1.15
**Place of residence**																
Urban®	1.00				1.00				1.00				1.00			
Rural	1.02	0.82	0.83	1.27	1.06	0.53	0.89	1.27	0.87	0.25	0.68	1.11	1.06	0.58	0.86	1.30

**Region**																
North®	1.00				1.00				1.00				1.00			
Central	1.72***	0.00	1.25	2.36	1.32*	0.05	1.00	1.74	1.81***	0.00	1.31	2.49	1.31	0.59	0.49	3.51
East	1.26	0.14	0.93	1.71	0.91	0.55	0.66	1.24	1.58***	0.01	1.14	2.19	2.79**	0.04	1.06	7.37
North East	3.31***	0.00	2.55	4.31	3.18***	0.00	2.39	4.23	3.15***	0.00	2.23	4.46	5.18***	0.00	1.98	13.55
West	0.39***	0.00	0.25	0.6	0.50***	0.00	0.35	0.73	0.81	0.42	0.47	1.37	0.79	0.66	0.27	2.31
South	0.65**	0.05	0.42	1	0.36***	0.00	0.26	0.49	0.66**	0.03	0.45	0.97	0.94	0.91	0.33	2.71
**Knowledge of adverse health effects of Smoked tobacco**																
No®	1.00				1.00				1.00				1.00			
Yes	0.67***	0.00	0.54	0.82	0.81***	0.01	0.70	0.95	1.24**	0.04	1.01	1.54	0.89	0.17	0.75	1.05
**Knowledge of adverse health effects of SLT**																
No®	1.00				1.00				1.00				1.00			
Yes	0.98	0.84	0.77	1.24	0.91	0.31	0.76	1.09	0.86	0.25	0.68	1.11	0.77***	0.01	0.65	0.92

Note: ® denotes reference category; * denotes p-values = <0.05; ** denotes p-value = <0.01; *** denotes p-value= <0.001; 95% CI denotes 95% Class Interval, OBC: Other Backward Classes
