# Supplementary material for: Caste and tobacco use: Decomposing inequalities using Global Adult Tobacco Survey, India
Source: PLoS One. 2026 Feb 11;21(2):e0341459. doi: 10.1371/journal.pone.0341459 (PMC12893575; doi:10.1371/journal.pone.0341459)
Supplement: S9 Table — (PDF) [file pone.0341459.s009.pdf]

S9 Table. Multivariate logistic regression decomposition estimates for caste differentials in smoked tobacco use among ST and Other (General/ OBC) social groups, 2016-17

Background characteristics	Due to Differences in Characteristics (E)					Due to the Difference in Coefficients (C)				
	Coefficient	p-value	95% CI		%	Coefficient	p-value	95% CI		%
**Age (in years)**										
15-18	1.000					1.000				
19-23	0.002	0.004	0.001	0.003	3.160	-0.001	0.393	-0.003	0.001	-1.570
24-30	0.003	0.002	0.001	0.005	5.700	-0.004	0.028	-0.008	0.000	-8.100
31-40	0.000	0.000	0.000	0.000	-0.360	-0.008	0.011	-0.013	0.002	-13.990
41-50	-0.002	0.000	-0.002	0.001	-3.060	-0.006	0.004	-0.011	0.002	-11.930
51-60	-0.003	0.000	-0.004	0.002	-5.470	-0.005	0.002	-0.008	0.002	-8.950
Over 60	-0.004	0.000	-0.006	0.002	-7.220	-0.004	0.010	-0.006	0.001	-6.600
**Sex**										
Female	1.000					1.000				
Male	0.007	0.000	0.006	0.009	13.570	-0.008	0.000	-0.012	0.003	-14.420
**Education**										
No formal schooling	1.000					1.000				
Below primary school or primary school completed	-0.002	0.006	-0.003	0.000	-3.060	0.002	0.007	0.001	0.004	3.860
Less than secondary school completed	-0.001	0.003	-0.001	0.000	-1.060	0.002	0.007	0.001	0.003	3.660
Secondary school completed	0.002	0.000	0.001	0.002	3.000	0.002	0.013	0.000	0.003	3.580
Greater than secondary school	0.006	0.000	0.004	0.009	11.330	0.006	0.000	0.003	0.010	11.780
**Marital status**										
Married	1.000					1.000				
Unmarried	0.000	0.923	-0.001	0.001	-0.090	0.001	0.365	-0.001	0.002	1.250
Widowed/Separated/Divorced	0.000	0.221	0.000	0.000	-0.300	0.000	0.747	-0.001	0.001	-0.240
**Occupation**										
Student	1.000					1.000				
Government employee	0.001	0.244	0.000	0.002	1.290	-0.001	0.067	-0.002	0.000	-1.440
Non-government employee	-0.002	0.225	-0.004	0.001	-2.790	-0.001	0.272	-0.003	0.001	-1.970
Daily Wage/Casual laborer	0.009	0.000	0.006	0.011	16.000	0.002	0.111	-0.001	0.005	4.230
Self-employed	0.003	0.000	0.001	0.004	5.400	0.000	0.915	-0.004	0.003	-0.340
Homemaker	-0.014	0.000	-0.020	0.009	-26.070	0.008	0.051	0.000	0.015	14.140
Retired/Unemployed and else	0.000	0.649	-0.001	0.001	0.280	-0.001	0.066	-0.002	0.000	-2.120
**Religion**										
Hindu	1.000					1.000				
Non-Hindu	0.008	0.024	0.001	0.015	14.370	-0.005	0.058	-0.011	0.000	-9.920
**Wealth quintile**										
Poorest	1.000					1.000				
Poorer	-0.002	0.000	-0.003	0.001	-4.290	-0.004	0.000	-0.006	0.002	-7.080
Middle	0.001	0.004	0.000	0.001	1.550	-0.001	0.048	-0.003	0.000	-2.550
Richer	0.001	0.233	-0.001	0.004	2.440	0.000	0.818	-0.002	0.002	-0.420
Richest	0.000	0.807	-0.003	0.003	-0.690	0.003	0.011	0.001	0.006	6.180
**Place of residence**										
Urban	1.000					1.000				
Rural	-0.004	0.023	-0.007	0.001	-7.350	-0.022	0.000	-0.034	0.010	-40.440
**Region**										
North	1.000					1.000				
Central	0.004	0.000	0.002	0.005	6.610	-0.001	0.507	-0.004	0.002	-1.660
East	0.005	0.000	0.003	0.006	8.410	-0.003	0.063	-0.005	0.000	-4.780
North East	0.002	0.858	-0.020	0.024	3.680	0.003	0.000	0.002	0.005	6.360
West	0.007	0.000	0.004	0.010	12.440	0.003	0.006	0.001	0.005	4.950
South	0.000	0.982	-0.008	0.008	0.170	0.006	0.000	0.003	0.009	10.930
**Knowledge of adverse health effects of smoke**										
No	1.000					1.000				
Yes	0.000	0.581	0.000	0.000	-0.010	-0.002	0.279	-0.005	0.001	-2.980
Overall	0.026	0.000	0.015	0.037	47.600	0.028	0.000	0.016	0.041	52.400
Constant						0.067	0.001	0.027	0.106	122.970
