# Supplementary material for: Caste and tobacco use: Decomposing inequalities using Global Adult Tobacco Survey, India
Source: PLoS One. 2026 Feb 11;21(2):e0341459. doi: 10.1371/journal.pone.0341459 (PMC12893575; doi:10.1371/journal.pone.0341459)
Supplement: S10 Table — (PDF) [file pone.0341459.s010.pdf]

S10 Table. Multivariate logistic regression decomposition estimates for caste differentials in smokeless tobacco use among ST and Other (General/ OBC) social groups, 2016-17

**Background characteristics**	**Due to Differences in Characteristics 'E'**					**Due to the Difference in Coefficients 'C'**				
	Coefficient	p-value	95% CI		%	Coefficient	p-value	95% CI		%
**Age (in years)**										
15-18	1.000					1.000				
19-23	0.002	0.000	0.001	0.003	1.38	-0.001	0.705	-0.004	0.003	-0.47
24-30	0.004	0.000	0.003	0.005	3.22	-0.001	0.769	-0.007	0.005	-0.72
31-40	0.000	0.000	0.000	0.000	-0.21	0.001	0.868	-0.008	0.010	0.60
41-50	-0.002	0.000	-0.002	0.001	-1.30	-0.003	0.443	-0.009	0.004	-2.06
51-60	-0.003	0.000	-0.003	0.002	-2.03	-0.003	0.249	-0.007	0.002	-2.13
Over 60	-0.003	0.000	-0.004	0.002	-2.55	-0.003	0.140	-0.008	0.001	-2.60
**Sex**										
Female	1.000					1.000				
Male	-0.001	0.000	-0.001	0.000	-0.63	-0.040	0.000	-0.049	0.030	-31.25
**Education**										
No formal schooling	1.000					1.000				
Below primary school or primary school completed	0.001	0.002	0.000	0.002	1.07	0.005	0.000	0.002	0.008	4.18
Less than secondary school completed	0.000	0.001	0.000	0.001	0.36	0.007	0.000	0.004	0.010	5.65
Secondary school completed	-0.001	0.056	-0.001	0.000	-0.40	0.007	0.000	0.004	0.010	5.48
Greater than secondary school	0.001	0.141	0.000	0.003	1.09	0.012	0.000	0.007	0.018	9.81
**Marital status**										
Married	1.000					1.000				
Unmarried	0.000	0.244	0.000	0.001	0.36	0.003	0.056	0.000	0.006	2.23
Widowed/Separated/Divorced	0.000	0.144	0.000	0.000	-0.10	-0.002	0.001	-0.004	0.001	-1.78
**Occupation**										
Student	1.000					1.000				
Government employee	0.001	0.02	0.000	0.002	0.84	-0.002	0.005	-0.004	0.001	-1.88
Non-government employee	-0.003	0.003	-0.005	0.001	-2.17	-0.007	0.000	-0.011	0.004	-5.78
Daily Wage/Casual laborer	0.005	0.000	0.003	0.006	3.57	-0.010	0.000	-0.016	0.005	-8.05
Self-employed	0.003	0.000	0.002	0.004	2.21	-0.010	0.003	-0.016	0.003	-7.76
Homemaker	-0.004	0.025	-0.007	0.000	-2.82	-0.016	0.012	-0.029	0.004	-12.92
Retired/Unemployed and else	0.001	0.000	0.000	0.001	0.74	-0.002	0.069	-0.004	0.000	-1.68
**Religion**										
Hindu	1.000					1.000				
Non-Hindu	-0.014	0.000	-0.019	0.009	-11.41	0.020	0.000	0.009	0.032	16.20
**Wealth quintile**										
Poorest	1.000					1.000				
Poorer	-0.001	0.085	-0.001	0.000	-0.52	-0.001	0.704	-0.003	0.002	-0.43
Middle	0.001	0.018	0.000	0.001	0.41	-0.002	0.107	-0.005	0.000	-1.63
Richer	0.003	0.000	0.001	0.005	2.50	0.000	0.962	-0.004	0.004	0.07
Richest	0.004	0.002	0.001	0.006	2.90	0.012	0.000	0.007	0.017	9.38
**Place of residence**										
Urban	1.000					1.000				
Rural	-0.003	0.048	-0.005	0.000	-2.02	-0.035	0.001	-0.056	0.013	-27.51
**Region**										
North	1.000					1.000				
Central	-0.007	0.000	-0.008	0.007	-5.76	0.014	0.015	0.003	0.026	11.34
East	-0.006	0.000	-0.007	0.006	-5.11	0.013	0.010	0.003	0.022	10.07
North East	0.126	0.000	0.110	0.142	99.69	0.001	0.729	-0.005	0.007	0.83
West	-0.012	0.000	-0.015	0.010	-9.73	0.006	0.142	-0.002	0.015	5.00
South	-0.022	0.000	-0.031	0.014	-17.75	0.020	0.021	0.003	0.036	15.51
**Knowledge of adverse health effects of SLT**										
No	1.000					1.000				
Yes	0.000	0.368	0.000	0.001	0.25	0.015	0.001	0.006	0.025	12.23
Overall	0.071	0.000	0.062	0.080	56.08	0.056	0.000	0.045	0.066	43.92
Constant						0.056	0.103	-0.011	0.122	44.00
