# Supplementary material for: Caste and tobacco use: Decomposing inequalities using Global Adult Tobacco Survey, India
Source: PLoS One. 2026 Feb 11;21(2):e0341459. doi: 10.1371/journal.pone.0341459 (PMC12893575; doi:10.1371/journal.pone.0341459)
Supplement: S11 Table — (PDF) [file pone.0341459.s011.pdf]

S11 Table. Multivariate logistic regression decomposition estimates for caste differentials in both tobaccos use among ST and Other social groups, 2016-17

Background characteristics	Due to Differences in Characteristics E					Due to the Difference in Coefficients C				
	Coefficient	p-value	95% CI		%	Coefficient	p-value	95% CI		%
**Age (in years)**										
15-18	1.000					1.000				
19-23	0.001	0.001	0.000	0.002	2.10	0.000	0.290	0.000	0.001	0.44
24-30	0.002	0.000	0.001	0.003	3.97	0.000	0.529	0.000	0.001	0.47
31-40	0.000	0.001	0.000	0.000	-0.20	0.000	0.520	-0.001	0.001	0.71
41-50	-0.001	0.001	-0.001	0.000	-1.35	0.000	0.571	-0.001	0.001	0.46
51-60	-0.001	0.001	-0.002	0.000	-2.22	0.000	0.519	0.000	0.001	0.36
Over 60	-0.001	0.158	-0.001	0.000	-1.27	0.000	0.832	0.000	0.001	0.11
**Sex**										
Female	1.000					1.000				
Male	0.002	0.000	0.001	0.002	3.29	-0.003	0.017	-0.005	0.000	-5.52
**Education**										
No formal schooling	1.000					1.000				
Below primary school or primary school completed	0.000	0.145	0.000	0.001	0.88	0.000	0.146	0.000	0.001	0.51
Less than secondary school completed	0.000	0.167	0.000	0.000	-0.27	0.000	0.586	0.000	0.000	0.15
Secondary school completed	0.000	0.010	0.000	0.001	0.99	0.000	0.157	0.000	0.001	0.53
Greater than secondary school	0.003	0.000	0.001	0.004	5.52	0.000	0.207	0.000	0.001	0.83
**Marital status**										
Married	1.000					1.000				
Unmarried	0.000	0.229	-0.001	0.000	-0.64	0.000	0.372	0.000	0.000	0.28
Widowed/Separated/Divorced	0.000	0.365	0.000	0.000	-0.12	0.000	0.420	0.000	0.000	-0.14
**Occupation**										
Student	1.000					1.000				
Government employee	0.000	0.141	0.000	0.001	0.93	0.000	0.188	0.000	0.000	-0.31
Non-government employee	-0.001	0.036	-0.002	0.000	-2.65	-0.001	0.069	-0.001	0.000	-1.09
Daily Wage/Casual laborer	0.001	0.032	0.000	0.003	3.03	-0.001	0.050	-0.002	0.000	-2.01
Self-employed	0.001	0.116	0.000	0.001	1.24	-0.001	0.051	-0.002	0.000	-2.48
Homemaker	-0.001	0.274	-0.004	0.001	-2.74	-0.001	0.280	-0.003	0.001	-2.07
Retired/Unemployed and else	0.000	0.023	0.000	0.001	0.77	0.000	0.139	-0.001	0.000	-0.51
**Religion**										
Hindu	1.000					1.000				
Non-Hindu	-0.001	0.630	-0.004	0.002	-1.53	0.000	0.988	-0.001	0.001	-0.02
**Wealth quintile**										
Poorest	1.000					1.000				
Poorer	0.000	0.134	-0.001	0.000	-0.79	0.000	0.582	0.000	0.000	-0.16
Middle	0.000	0.076	0.000	0.001	0.55	0.000	0.777	0.000	0.000	-0.08
Richer	0.000	0.664	-0.001	0.001	0.49	0.000	0.244	0.000	0.001	0.55
Richest	0.001	0.278	-0.001	0.002	1.77	0.000	0.185	0.000	0.001	0.82
**Place of residence**										
Urban	1.000					1.000				
Rural	0.001	0.579	-0.001	0.002	1.02	0.000	0.833	-0.002	0.002	0.44
**Region**										
North	1.000					1.000				
Central	0.000	0.491	-0.001	0.001	-0.78	0.000	0.774	-0.001	0.001	-0.25
East	-0.001	0.008	-0.002	0.000	-2.16	0.001	0.031	0.000	0.001	1.30
North East	0.038	0.000	0.022	0.053	77.63	0.000	0.216	0.000	0.001	0.52
West	0.001	0.717	-0.002	0.003	1.07	0.000	0.296	0.000	0.001	0.68
South	0.000	0.978	-0.007	0.007	0.21	0.001	0.134	0.000	0.002	1.78
**Knowledge of adverse health effects of SLT**										
No										
Yes	0.001	0.006	0.000	0.001	1.53	-0.001	0.138	-0.002	0.000	-1.80
**Knowledge of adverse health effects of smoke**										
No	1.000					1.000				
Yes	0.000	0.172	0.000	0.000	-0.02	0.001	0.154	0.000	0.001	1.12
Overall	0.044	0.000	0.039	0.049	90.25	0.005	0.066	0.000	0.010	9.75
Constant						0.007	0.199	-0.004	0.017	14.12
