# Supplementary material for: Caste and tobacco use: Decomposing inequalities using Global Adult Tobacco Survey, India
Source: PLoS One. 2026 Feb 11;21(2):e0341459. doi: 10.1371/journal.pone.0341459 (PMC12893575; doi:10.1371/journal.pone.0341459)
Supplement: S12 Table — (PDF) [file pone.0341459.s012.pdf]

S12 Table. Multivariate logistic regression decomposition estimates for caste differentials in total tobacco use among ST and SC group population, 2016-17

**Background characteristics**	**Due to Differences in Characteristics E**					**Due to the Difference in Coefficients C**				
	Coefficient	P-value	95% CI		%	Coefficient	P-value	95% CI		%
**Age (in years)**										
15-18	1.000					1.000				
19-23	0.002	0.000	0.002	0.003	1.560	0.001	0.457	-0.002	0.005	0.900
24-30	0.003	0.000	0.002	0.004	1.860	0.002	0.539	-0.005	0.010	1.510
31-40	-0.003	0.000	-0.004	0.003	-2.070	0.002	0.696	-0.008	0.012	1.260
41-50	0.000	0.000	0.000	0.000	0.260	-0.003	0.334	-0.010	0.003	-2.020
51-60	-0.002	0.000	-0.002	0.001	-1.080	-0.005	0.024	-0.009	0.001	-3.080
Over 60	-0.003	0.000	-0.003	0.002	-1.600	-0.006	0.006	-0.009	0.002	-3.490
**Sex**										
Female	1.000					1.000				
Male	0.009	0.000	0.008	0.009	5.510	-0.029	0.000	-0.037	0.020	-18.250
**Education**										
No formal schooling	1.000					1.000				
Below primary school or primary school completed	0.001	0.046	0.000	0.001	0.460	0.007	0.001	0.003	0.011	4.350
Less than secondary school completed	0.000	0.729	0.000	0.001	0.050	0.005	0.001	0.002	0.008	3.270
Secondary school completed	0.000	0.004	0.000	0.000	-0.070	0.005	0.000	0.002	0.008	3.240
Greater than secondary school	-0.002	0.000	-0.002	0.001	-1.010	0.007	0.000	0.003	0.011	4.490
**Marital status**										
Married	1.000					1.000				
Unmarried	0.000	0.752	-0.001	0.001	0.130	0.003	0.082	0.000	0.006	1.910
Widowed/Separated/Divorced	-0.001	0.035	-0.001	0.000	-0.430	-0.001	0.180	-0.003	0.001	-0.730
**Occupation**										
Student	1.000					1.000				
Government employee	0.002	0.007	0.001	0.004	1.570	0.000	0.504	-0.002	0.001	-0.310
Non-government employee	-0.004	0.000	-0.005	0.002	-2.230	-0.003	0.038	-0.007	0.000	-2.170
Daily Wage/Casual laborer	-0.011	0.000	-0.014	0.009	-7.200	-0.004	0.457	-0.014	0.006	-2.440
Self-employed	0.015	0.000	0.011	0.019	9.730	-0.003	0.294	-0.008	0.002	-1.710
Homemaker	-0.011	0.000	-0.016	0.007	-7.180	0.001	0.841	-0.012	0.015	0.880
Retired/Unemployed and else	0.003	0.000	0.001	0.004	1.640	0.000	0.711	-0.003	0.002	-0.250
**Religion**										
Hindu	1.000					1.000				
Non-Hindu	-0.021	0.000	-0.032	0.010	-13.300	-0.017	0.036	-0.034	0.001	-11.090
**Wealth quintile**										
Poorest	1.000					1.000				
Poorer	0.001	0.000	0.000	0.001	0.400	-0.006	0.013	-0.010	0.001	-3.550
Middle	0.002	0.000	0.001	0.003	1.240	0.001	0.664	-0.002	0.004	0.400
Richer	0.005	0.000	0.003	0.006	2.910	0.003	0.086	0.000	0.007	2.160
Richest	-0.001	0.000	-0.002	0.001	-0.810	0.004	0.003	0.001	0.007	2.510
**Place of residence**										
Urban	1.000					1.000				
Rural	-0.003	0.003	-0.004	0.001	-1.610	-0.022	0.074	-0.047	0.002	-14.130
**Region**										
North	1.000					1.000				
Central	-0.007	0.000	-0.010	0.005	-4.740	0.004	0.126	-0.001	0.009	2.550
East	-0.014	0.000	-0.018	0.010	-8.980	0.007	0.006	0.002	0.012	4.490
North East	0.170	0.000	0.142	0.197	107.810	-0.001	0.311	-0.004	0.001	-0.900
West	0.000	0.020	0.000	0.000	-0.160	0.000	0.934	-0.003	0.002	-0.060
South	-0.009	0.043	-0.019	0.000	-6.000	0.009	0.005	0.003	0.015	5.620
**Knowledge of adverse health effects of SLT**										
No	1.000					1.000				
Yes	0.002	0.000	0.001	0.003	1.400	-0.001	0.833	-0.012	0.010	-0.750
**Knowledge of adverse health effects of smoke**										
No	1.000					1.000				
Yes	0.000	0.673	-0.001	0.000	-0.060	0.008	0.025	0.001	0.015	5.050
Overall	0.123	0.000	0.108	0.138	77.97	0.035	0.000	0.017	0.053	22.03
Constant						0.067	0.049	0.000	0.133	42.35
